# Supplementary figures and images for: Patient knowledge in anaesthesia: Psychometric development of the RAKQ–The Rotterdam anaesthesia Knowledge questionnaire
Source: PLoS One. 2024 Jul 12;19(7):e0299052. doi: 10.1371/journal.pone.0299052 (PMC11244777; doi:10.1371/journal.pone.0299052)

Online Supporting Information Figure S4. Scree plots following Modified Parallel Analysis.

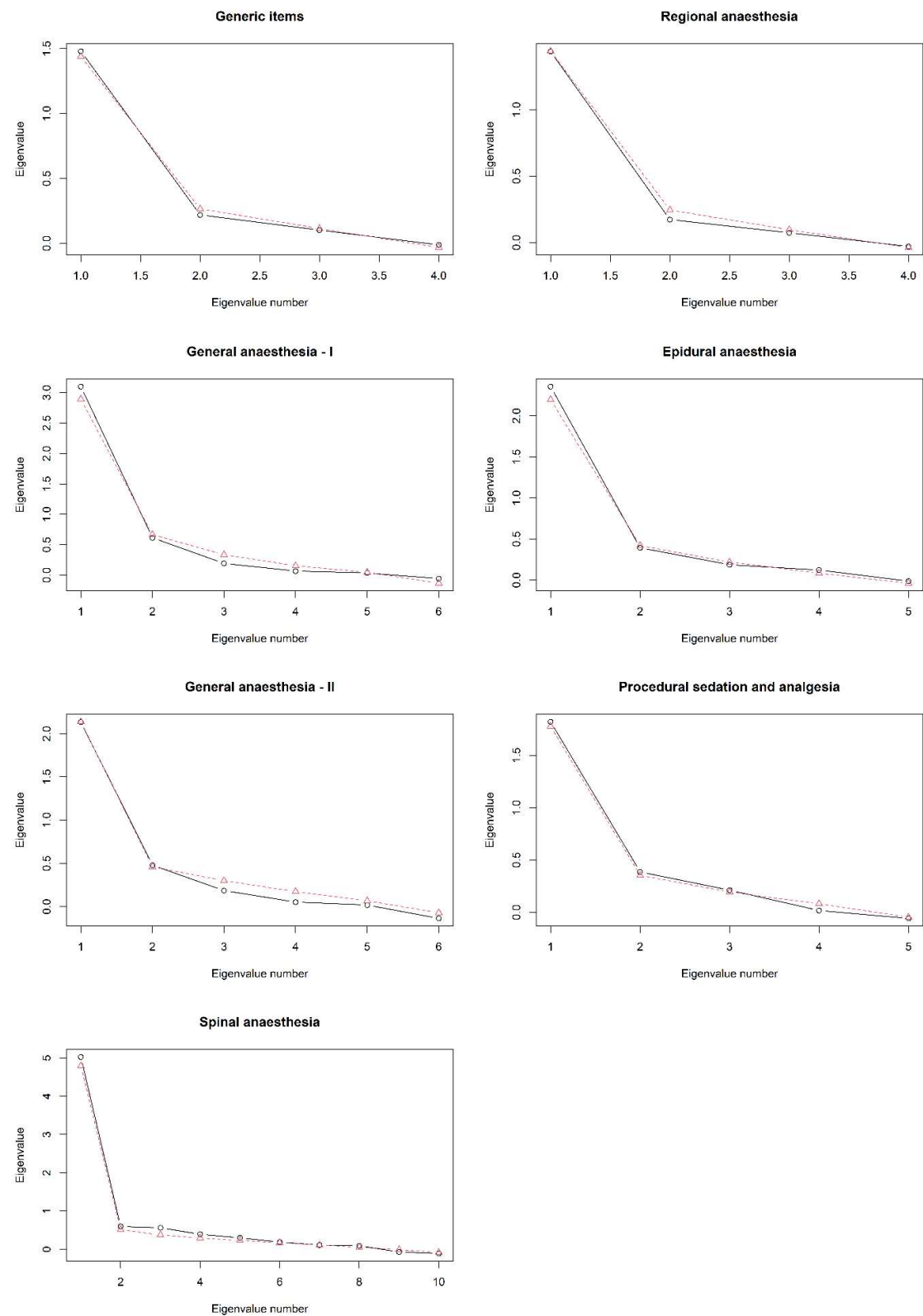

Supplement: S1 Fig — (PDF) [file pone.0299052.s001.pdf]
