# Supplementary material for: Patient knowledge in anaesthesia: Psychometric development of the RAKQ–The Rotterdam anaesthesia Knowledge questionnaire
Source: PLoS One. 2024 Jul 12;19(7):e0299052. doi: 10.1371/journal.pone.0299052 (PMC11244777; doi:10.1371/journal.pone.0299052)
Supplement: S1 File — (DOCX) [file pone.0299052.s002.docx]

**S2 Document. Alterations in the items between the two study sites**

**Items that were altered between administration in the Erasmus
MC and Albert Schweitzer Hospital (ASZ**).

**GEN10:**

**Erasmus MC:** You may drink milk 6 hours before any form of anaesthesia (general anaesthesia, regional anaesthesia, spinal anaesthesia).

1. True
2. False
3. I do not know

**ASZ:** You may drink milk 4 hours before any form of anaesthesia (general anaesthesia, regional anaesthesia, spinal anaesthesia).

1. True
2. False
3. I do not know

*Reason: 6 hours is exactly on the limit of the fasting period, 4 hours is more clearly within the fasting period.*

**GEN11:**

**Erasmus MC:** Which of the fluids stated below may you drink up to 2 hours before the operation?

1. Freshly squeezed orange juice
2. Milk
3. Tea
4. I do not know

**ASZ:** Which of the fluids stated below may you drink **up to** 2 hours before the operation?

1. Freshly squeezed orange juice
2. Milk
3. Tea
4. I do not know

*Reason: emphasized “up to” to make the question more clear.*

**GEN2:**

**Erasmus MC:** Patients are generally seen in advance of the surgery by the same anaesthetist who administers the anaesthesia on the day of the surgery.

1. True
2. False
3. I do not know

**ASZ:** Patients are generally seen in advance of the surgery by the **same** anaesthetist who administers the anaesthesia on the day of the surgery.

1. True
2. False
3. I do not know

*Reason*: *emphasized “same” to make the question more clear.*

**GA2:**

**Erasmus MC**: What do you notice from the breathing tube placed in your mouth during the operation?

1. It is often painful because the tube is placed before the start of general anesthesia
2. Very little, maybe a brief period of throat pain and hoarseness after the surgery
3. This is placed under local anesthesia before the start of general anesthesia
4. I do not know

**ASZ:** What do you notice, after the operation, from the breathing tube placed in your mouth during the operation?

1. Often, there is intense pain in the throat
2. Sometimes there is a brief period of sore throat and hoarseness
3. Often your voice is lost for several days
4. I do not know

*Reason: question was made a bit more clear, and the answers were modified to better fit the question concerning complaints after the operation.*

**GA3:**

**Erasmus MC:** How does an anaesthetist administer medication that puts a person under anaesthesia?

1. By giving you a tablet /pill
2. By giving you spinal anesthesia ( an injection in the back)
3. By giving you an injection in a vein through a line.
4. I do not know

**ASZ:** How is the medication that puts a person under anaesthesia administered?

1. By giving you a tablet /pill
2. By giving you spinal anesthesia ( an injection in the back)
3. By giving you an injection in a vein through a line.
4. I do not know

*Reason: removed the word ‘anaesthetist’ from the question, trying not to give away the answer to GA5.*

**GA8:**

**Erasmus MC:** Patients usually wake up rapidly after the operation.

1. True
2. False
3. I do not know

**ASZ:** Patients usually wake up within half an hour after the operation.

1. True
2. False
3. I do not know

*Reason: made the question more concrete by specifying a time frame.*

**GA10:**

**Erasmus MC:** How big is the chance that your teeth could be damaged during the placement of the breathing tube?

1. Low
2. High
3. I do not know

**ASZ:** Sometimes, your teeth can be damaged during the placement of the breathing tube.

1. True
2. False
3. I do not know

*Reason: changed question asking about chances to a true/false question in line with most questions in the questionnaire.*
