# Supplementary material for: Patient knowledge in anaesthesia: Psychometric development of the RAKQ–The Rotterdam anaesthesia Knowledge questionnaire
Source: PLoS One. 2024 Jul 12;19(7):e0299052. doi: 10.1371/journal.pone.0299052 (PMC11244777; doi:10.1371/journal.pone.0299052)
Supplement: S2 File — (DOCX) [file pone.0299052.s003.docx]

**Online Supporting Information Document S6.** Differential Item Functioning

***Generic items*** Items GEN3 and GEN11 showed non-uniform DIF for age. The younger population was more likely to answer item GEN3, about premedication in case of anxiety, correctly, and item GEN11, about fluid intake preoperatively, incorrectly. The effect of DIF on the total score was at most 0.4 SD’s, smaller than the SEM (0.75).

***General anaesthesia – I*** Item GA2 showed uniform DIF for the hospital. The answer options for this item, regarding placement of the breathing tube, were adjusted before the survey was administered in the ASZ for clarity. The ASZ population was more likely to correctly answer this question. The effect of DIF on the total score was at most 0.15 SD’s, smaller than the SEM (0.72). Items GA4 and GA5 showed non-uniform DIF for age. The older population was more likely to answer item GA4, about the recovery room, and GA5, about who administers the general anaesthesia, correctly. The effect of DIF on the total score was at most 0.2 SD’s, smaller than the SEM (0.72). Item GA4 showed uniform DIF for level of education. The population with a lower education was more likely to answer item GA4, about the recovery room, and GA5, about who administers the general anaesthesia, correctly. The effect of DIF on the total score was at most 0.2 SD’s, smaller than the SEM (0.72).

***General anaesthesia – II*** Item GA10 showed uniform DIF and items GA16 and GA12 showed non-uniform DIF for the hospital. The EMC population was more likely to answer item GA10, about damage to the teeth during intubation, and GA16, about postoperative cognitive impairment, correctly. The ASZ population was more likely to correctly answer item GA12, about postoperative nausea. Items GA6 and GA14 showed non-uniform DIF for level of education. The population with a lower education level and higher ability levels was more likely to correctly answer item GA6 about nerve damage due to positioning. The population with a higher education level and lower ability levels was more likely to correctly answer GA14 about loose teeth.

***Spinal anaesthesia*** Item SA11 showed uniform DIF for age. The younger population was more likely to correctly answer item SA11 about the timing of hospital discharge after spinal anaesthesia.

***Regional anaesthesia*** Item RA2 showed uniform DIF for age. The younger population was more likely to correctly answer item RA2 about the use of ultrasonography to facilitate regional blockade.

***Epidural anaesthesia*** Item EA1 showed uniform DIF for the use of epidural analgesia. The population in which epidural analgesia was discussed preoperatively was more likely to correctly answer item EA1 about the timing of epidural catheter removal.

***Procedural sedation and analgesia*** No items showed DIF.
