# Supplementary material for: Patient knowledge in anaesthesia: Psychometric development of the RAKQ–The Rotterdam anaesthesia Knowledge questionnaire
Source: PLoS One. 2024 Jul 12;19(7):e0299052. doi: 10.1371/journal.pone.0299052 (PMC11244777; doi:10.1371/journal.pone.0299052)
Supplement: S1 Table — (DOCX) [file pone.0299052.s004.docx]

**Online Supporting Information Table S1**. Glossary of terms

| Computerised-Adaptive Test (CAT) | A computer-administered test in which, after the first item, the presentation of items is determined by individuals’ responses to previous ones. The selection of items is adapted to prior estimates of the respondents’ level of the construct/domain being measured [1]. |
| --- | --- |
| Confirmatory factor analysis (CFA) | Factor analysis that requires the researcher to have specific expectations regarding the number and nature of underlying constructs. CFA explicitly tests the fit of factor models [2]. |
| Content validity | The degree to which elements of an assessment instrument are relevant to and representative of the targeted construct for a particular assessment purpose [3]. |
| Differential item functioning (DIF) | The degree to which respondents from different groups answer differently on certain items while they ought to have the same ability level [4]. Uniform DIF: The direction of the difference is the same across all trait levels. Non-uniform DIF: The direction changes across different trait levels. |
| Exploratory factor analysis (EFA) | Factor analysis without the need for the researcher to declare any expectations regarding the number or nature of underlying constructs [2]. |
| Face validity | The degree to which users judge that the items of an assessment instrument are appropriate for the targeted construct and assessment objectives [3]. |
| Factor Analysis | A regression model in which observed standardised variables are regressed to unobserved (latent) factors. It is a data-reduction technique used to understand the latent structure of a set of items [5]. |
| Factor loadings | Regression coefficients that are also correlations, representing the loading of each observed variable on each factor [5]. |
| (item and test) Information | In IRT, each response pattern results in a different theta (i.e. level of ability) and a different associated standard error. The information of an item or test represents the degree of certainty about the respondent’s level of ability (theta). This is a complementary perspective to the standard error of the estimate, which indicates the degree of uncertainty about the level of ability [6]. |
| Invariant Item Ordering (IIO) | The degree to which the relative ordering of the items in terms of the latent trait/domain is invariant across respondents [7]. The ordering of items does not depend on who filled in the items or which items were filled in in which sequence. |
| Item fit | The degree to which the items consistently order respondents on the latent variable [7]. |
| Item Response Function (IRF) | The IRF represents the probability (P) of a respondent answering a certain item in a certain way (e.g. correct or incorrect) as a function of the ability (e.g. knowledge of a specific domain, represented in theta) of the individual (. The IRF can have one (difficulty), two (difficulty and discrimination), or three (difficulty, discrimination, and guessing) parameters (1PL or Rasch, and 2PL and 3PL models, respectively). |
| Item Response Theory (IRT) | A family of models that describe, in probabilistic terms, the relationship between a person’s response to an item from a scale and his or her level of ability (i.e. knowledge on a specific domain) on the latent construct that the scale measures [4]. |
| Latent Factor / Traits / Construct / Ability / Theta | An indirectly measured dimension underlying a set of items (scale); for example, fear can be measured by XX items. |
| Local (in)dependence | The degree to which significant correlations between item responses do or do not exist, when the underlying dominant ability that explains a person’s response to an item is controlled. |
| Monotonicity | The degree to which the relative ordering of respondents in terms of the latent trait/domain is invariant across items [7]. |
| Person fit | The degree to which an individual’s response pattern is unusual or inconsistent with the model used to characterise their ability level [6]. |
| Scalability | The degree to which items can be used to distinguish among respondents in terms of the latent variable [7]. |
| Unidimensionality | The underlying scale of a set of items measures one trait/ability, such that the level of the underlying ability gives rise to the response to the items of the scale [4]. |

**References**

1. Cook KF, O'Malley KJ, Roddey TS. Dynamic Assessment of Health Outcomes: Time to Let the CAT Out of the Bag? *Health Services Research* 2005; **40:** 1694-711.

2. Thompson B. *Exploratory and confirmatory factor analysis: Understanding concepts and applications*. Washington, DC, US: American Psychological Association, 2004.

3. Haynes SN, Richard DCS, Kubany ES. Content validity in psychological assessment: A functional approach to concepts and methods. *Psychological Assessment* 1995; **7:** 238-47.

4. Reeve BB, Hays RD, Bjorner JB, et al. Psychometric evaluation and calibration of health-related quality of life item banks: plans for the Patient-Reported Outcomes Measurement Information System (PROMIS). *Med Care* 2007; **45:** S22-31.

5. Boateng GO, Neilands TB, Frongillo EA, Melgar-Quinonez HR, Young SL. Best Practices for Developing and Validating Scales for Health, Social, and Behavioral Research: A Primer. *Front Public Health* 2018; **6:** 149.

6. de Ayala RJ. *The theory and practice of item response theory*. New York, NY, US: Guilford Press, 2009.

7. Wind S. Examining the Psychometric Quality of Multiple-Choice Assessment Items using Mokken Scale Analysis. *Journal of applied measurement* 2016; **17:** 142-65.
