# Supplementary material for: Patient knowledge in anaesthesia: Psychometric development of the RAKQ–The Rotterdam anaesthesia Knowledge questionnaire
Source: PLoS One. 2024 Jul 12;19(7):e0299052. doi: 10.1371/journal.pone.0299052 (PMC11244777; doi:10.1371/journal.pone.0299052)
Supplement: S2 Table — (DOCX) [file pone.0299052.s005.docx]

| **Online Supporting Information Table S3.** Comparison of nested multidimensional Item Response Theory models for exploratory factor analysis. In bold the chosen model and the optimal fit parameters. | | | | | |
| --- | --- | --- | --- | --- | --- |
|  | | | | | |
| Model – n dimensions | log-Lik | AIC | BIC | Comparing models | |
| *Generic items* | |  |  |  | |
| 1 | -3794.23 | 7636.45 | **7741.04** |  | |
| 2 | -3775.98 | 7621.96 | 7774.48 | mod1 vs. mod2; ChiSq= 36.493; df=11; p=0.000 | |
| **3** | -3749.68 | **7589.35** | 7785.45 | mod2 vs. mod3; ChiSq=52.606; df=10; p=0.000 | |
| 4 | -3745.37 | 7598.74 | 7834.07 | mod3 vs. mod4; ChiSq=8.607; df=9; **p=0.474** | |
| *General anaesthesia* | |  |  |  | |
| 1 | -4147.03 | 8358.05 | **8497.50** |  | |
| 2 | -4121.05 | 8336.10 | 8540.92 | mod1 vs. mod2; ChiSq=51.954; df=15; p=0.000 | |
| 3 | -4103.13 | 8328.25 | 8594.08 | mod2 vs. mod3; ChiSq=35.848; df=14; p=0.001 | |
| **4** | -4085.37 | **8318.74** | 8641.22 | mod3 vs. mod4; ChiSq=35.511; df=13; p=0.001 | |
| 5 | -4077.25 | 8326.51 | 8701.28 | mod5 vs. mod4; ChiSq=16.233; df=12; **p=0.181** | |
| *Spinal anaesthesia* | |  |  |  | |
| 1 | -3956.73 | 7965.46 | **8078.76** |  | |
| **2** | -3922.19 | **7920.38** | 8085.97 | mod1 vs. mod2; ChiSq=69.082; df=12; p=0.000 | |
| 3 | -3911.90 | 7921.79 | 8135.32 | mod2 vs. mod3; ChiSq=20.585; df=11; p=0.038 | |
| 4 | -3908.12 | 8004.50 | 8191.35 | mod3 vs. mod4; ChiSq=7.555; df=10; **p=0.672** | |
| *Regional anaesthesia* | |  |  |  | |
| 1 | -2132.79 | 4293.57 | **4354.58** |  | |
| **2** | -2124.52 | **4289.03** | 4376.19 | mod1 vs. mod2; ChiSq=16.540; df=6; p=0.011 | |
| 3 | -2124.50 | 4299.00 | 4407.95 | mod2 vs. mod3; ChiSq=0.031; df=5; **p=1.000** | |
| *Epidural anaesthesia* | |  |  |  | |
| 1 | -1865.93 | 3755.86 | **3808.16** |  | |
| **2** | -1854.78 | **3743.56** | 3817.65 | mod1 vs. mod2; ChiSq=22.300; df=5; p=0.000 | |
| 3 | -1855.51 | 3753.01 | 3844.52 | mod2 vs. mod3; ChiSq=-1.447; df=4; **p=1.000** | |
| *Procedural sedation and analgesia* | |  |  |  | |
| 1 | -2001.79 | 4027.58 | **4079.88** |  | |
| **2** | -1990.35 | **4014.70** | 4088.78 | mod1 vs. mod2; ChiSq=22.882; df=5; p=0.000 | |
| 3 | -1989.17 | 4020.33 | 4111.84 | mod2 vs. mod3; ChiSq=2.371; df=4; **p=0.668** | |
|  | | | | |  |
