# Supplementary material for: Patient knowledge in anaesthesia: Psychometric development of the RAKQ–The Rotterdam anaesthesia Knowledge questionnaire
Source: PLoS One. 2024 Jul 12;19(7):e0299052. doi: 10.1371/journal.pone.0299052 (PMC11244777; doi:10.1371/journal.pone.0299052)
Supplement: S3 Table — (DOCX) [file pone.0299052.s006.docx]

| **Online Supporting Information Table S5.** Comparison of 1-, 2- and 3-PL Item Response Theory models. In bold the chosen model and the optimal fit parameters. | | | | |
| --- | --- | --- | --- | --- |
|  | | | | |
| IRT Model | log-Lik | AIC | BIC | Comparing models |
| *Generic items* | |  |  |  |
| **1-PL** | -1187.72 | **2385.43** | **2407.22** |  |
| 2-PL | -1185.66 | 2387.31 | 2422.18 | mod1 vs. mod2; ChiSq=4.122; df=3; **p=0.249** |
| 3-PL | -1185.64 | 2395.28 | 2447.57 | mod2 vs. mod3; ChiSq=0.035; df=4; p=1 |
| *General anaesthesia – I* |  |  |  |  |
| 1-PL | -1234.30 | 2482.61 | **2513.11** |  |
| **2-PL** | -1227.30 | **2478.60** | 2530.89 | mod1 vs. mod2; ChiSq=14.011; df=5; p=0.016 |
| 3-PL | -1226.19 | 2488.38 | 2566.82 | mod2 vs. mod3; ChiSq=2.215; df=6; **p=0.899** |
| *General anaesthesia – II* |  |  |  |  |
| **1-PL** | -1733.30 | **3480.59** | **3511.10** |  |
| 2-PL | -1729.26 | 3482.51 | 3534.81 | mod1 vs. mod2; ChiSq=8.049; df=5; **p=0.152** |
| 3-PL | -1729.25 | 3494.50 | 3572.94 | mod2 vs. mod3; ChiSq=0.012; df=6; p=1 |
| *Spinal anaesthesia* |  |  |  |  |
| 1-PL | -3084.58 | 6191.16 | **6239.10** |  |
| **2-P**L | -3071.01 | **6182.02** | 6269.17 | mod1 vs. mod2; ChiSq=27.147; df=9; p=0.001 |
| 3-PL | -3071.01 | 6202.02 | 6332.75 | mod2 vs. mod3; ChiSq=0.000; df=10; **p=1** |
| *Regional anaesthesia* | |  |  |  |
| **1-PL** | -1262.94 | **2535.87** | **2557.66** |  |
| 2-PL | -1261.78 | 2539.57 | 2574.43 | mod1 vs. mod2; ChiSq=2.301; df=3; p=**0.512** |
| 3-PL | -1261.79 | 2547.57 | 2599.87 | mod2 vs. mod3; ChiSq=-0.005 ; df=4; p=1 |
| *Epidural anaesthesia* | |  |  |  |
| 1-PL | -1592.68 | 3197.36 | **3223.51** |  |
| **2-PL** | -1580.99 | **3181.99** | 3225.57 | mod1 vs. mod2; ChiSq=23.369; df=4; p=0 |
| 3-PL | -1580.99 | 3191.99 | 3257.36 | mod2 vs. mod3; ChiSq=0.000 ;df=5; **p=1** |
| *Procedural sedation and analgesia* | |  |  |  |
| 1-PL | -1639.70 | 3291.40 | **3317.55** |  |
| **2-PL** | -1633.79 | **3287.59** | 3331.17 | mod1 vs. mod2; ChiSq=11.818; df=4; p=0.019 |
| 3-PL | -1631.65 | 3293.30 | 3358.66 | mod2 vs. mod3; ChiSq=4.290; df=5; **p=0.508** |
