# Supplementary material for: Patient knowledge in anaesthesia: Psychometric development of the RAKQ–The Rotterdam anaesthesia Knowledge questionnaire
Source: PLoS One. 2024 Jul 12;19(7):e0299052. doi: 10.1371/journal.pone.0299052 (PMC11244777; doi:10.1371/journal.pone.0299052)
Supplement: S5 Table — (DOCX) [file pone.0299052.s008.docx]

| **Online Supporting Information Table S7.** Final questionnaires English Translation. | |
| --- | --- |
|  |  |
| ***Generic items*** | |
| GEN2 | Patients are generally seen in advance of the surgery by the same anaesthetist who administers the anaesthesia on the day of the surgery. |
|  | 1. *True* 2. *False* 3. *I do not know* |
| GEN3 | What can anaesthetists do to reduce anxiety? |
|  | - 1. *Give the patient a tablet to calm them*   2. *Put the patient under anaesthesia on the ward*   3. *The patient can reschedule the operation for another day*   4. *I do not know* |
| GEN8 | What must you do with your usual daily medications? |
|  | 1. *The anaesthetist will discuss and arrange this with you. Usually you may continue using your all medication* 2. *Don’t use any medication, bring all medication with you to the hospital to take it after the operation* 3. *You must consult your own general practitioner about this* 4. *I do not know* |
| GEN11 | Which of the fluids stated below may you drink up to 2 hours before the operation? |
|  | 1. *Freshly squeezed orange juice* 2. *Milk* 3. *Tea* 4. *I do not know* |
|  | |
| ***General anaesthesia - I*** | |
| GA2 | What do you notice from the breathing tube placed in your mouth during the operation? |
|  | 1. *It is often painful because the tube is placed before the start of general anaesthesia* 2. *Very little, maybe a brief period of throat pain and hoarseness after the surgery* 3. *This is placed under local anaesthesia before the start of general anaesthesia* 4. *I do not know* |
| GA3 | How does an anaesthetist administer medication that puts a person under anaesthesia? |
|  | 1. *By giving you a pill* 2. *By giving you spinal anaesthesia (an injection in the back)* 3. *By giving you an injection in a vein through a line.* 4. *I do not know* |
| GA4 | Where do most patients wake up after general anaesthesia? |
|  | 1. *In the operating room, recovery room or intensive care unit* 2. *On the surgical ward where the patient was before surgery* 3. *At home* 4. *I do not know* |
| GA5 | Who administers general anaesthesia? |
|  | 1. *A nurse* 2. *An anaesthetist* 3. *A surgeon* 4. *I do not know* |
| GA7 | To insert the breathing tube, the anaesthetist makes a small incision in the wind pipe and reseals the opening at the end of the operation. |
|  | 1. *True* 2. *False* 3. *I do not know* |
| GA9 | When is the breathing tube placed? |
|  | 1. *Before the patient goes to sleep* 2. *After the patient goes to sleep and before the operation begins* 3. *After the patient goes to sleep and the after operation has started* 4. *I do not know* |
|  | |
| ***General anaesthesia - II*** | |
| GA6 | Under general anaesthesia, it is possible to develop a damaged nerve from being in a particular position for too long. |
|  | 1. *True* 2. *False* 3. *I do not know* |
| GA10 | Sometimes, your teeth can be damaged during the placement of the breathing tube. |
|  | 1. *True* 2. *False* 3. *I do not know* |
| GA14 | Patients must tell the anaesthetists if they have loose teeth. |
|  | 1. *True* 2. *False* 3. *I do not know* |
| GA16 | After an operation under general anaesthesia, a patient’s ability to concentrate can be reduced for a short period of time. |
|  | 1. *True* 2. *False* 3. *I do not know* |
| GA12 | Does every patient experience nausea after general anaesthesia? |
|  | 1. *No, only patients vulnerable to nausea* 2. *Yes, it is unavoidable* 3. *No, this rarely happens* 4. *I do not know* |
| GA15 | Can general anaesthesia cause dementia? |
|  | 1. *Yes* 2. *No* 3. *I do not know* |
|  |  |
| ***Spinal anaesthesia*** | |
| SA1 | What part of the body is numbed during spinal anaesthesia? |
|  | 1. *From the belly button to the toes* 2. *From the neck to the belly button* 3. *The entire body* 4. *It can differ, often a combination of the first three answers* 5. *I do not know* |
| SA2 | What are the possible side effects of spinal anaesthesia? |
|  | 1. *Loss of smell* 2. *Sleepy* 3. *Headache* 4. *I do not know* |
| SA4 | A spinal injection is more painful than a regular injection in the arm. |
|  | 1. *True* 2. *False* 3. *I do not know* |
| SA5 | How long before spinal anaesthesia wears off? |
|  | 1. *Less than 1 hour* 2. *1 to 6 hours* 3. *6 to 12 hours* 4. *I do not know* |
| SA6 | Are mild tingling sensations normal during spinal anaesthesia placement? |
|  | 1. *Yes, they are* 2. *No, they are not* 3. *I do not know* |
| SA7 | Important vital signs (e.g. blood pressure and heart rate) are measured during spinal anaesthesia, similar to general anaesthesia. |
|  | 1. *True* 2. *False* 3. *I do not know* |
| SA9 | During an operation under spinal anaesthesia, it is possible to also receive sedation. |
|  | 1. *True* 2. *False* 3. *I do not know* |
| SA11 | Patients are discharged home faster after spinal anaesthesia than after general anaesthesia. |
|  | 1. *True* 2. *False* 3. *I do not know* |
| SA12 | After the operation, as soon as feeling and strength have returned to the legs after spinal anaesthesia, patients are allowed to drive home themselves. |
|  | 1. *True* 2. *False* 3. *I do not know* |
| SA13 | What action does the anaesthetist take if spinal anaesthesia fails after one or more attempts? |
|  | 1. *Conducts general anaesthesia* 2. *Cancels the operation* 3. *This situation never arises* 4. *I do not know* |
|  |  |
| ***Regional anaesthesia*** | |
| RA2 | Which tool can anaesthetists use to administer regional anaesthesia? |
|  | 1. *An infrared thermometer* 2. *An X-Ray machine* 3. *An ultrasound machine* 4. *I do not know* |
| RA3 | Regional anaesthesia can work for longer than 12 hours. |
|  | 1. *True* 2. *False* 3. *I do not know* |
| RA6 | During regional anaesthesia, the affected limb is not only numb, but the patient is also unable to move it. |
|  | 1. *True* 2. *False* 3. *I do not know* |
| RA7 | The chance of nausea after regional anaesthesia is lower than that after general anaesthesia? |
|  | 1. *True* 2. *False* 3. *I do not know* |
|  |  |
| ***Epidural anaesthesia*** | |
| EA1 | The tube for pain medication is removed immediately after the operation. |
|  | 1. *True* 2. *False* 3. *I do not know* |
| EA3 | Permanent nerve damage after epidural anaesthesia is very rare. |
|  | 1. *True* 2. *False* 3. *I do not know* |
| EA4 | What is the advantage of epidural anaesthesia over the use of pain medication in drips? |
|  | 1. *Less drowsiness and nausea with epidural anaesthesia* 2. *Earlier discharge from hospital with epidural anaesthesia* 3. *There is no advantage* 4. *I do not know* |
| EA5 | If a patient with epidural anaesthesia cannot move their legs, what must he/she do? |
|  | 1. *Alert the doctor on the ward the next day* 2. *Alert the nurse immediately* 3. *Nothing, not being able to move your legs is to be expected* 4. *I do not know* |
| EA6 | What are the options if placement of the epidural was not successful? |
|  | 1. *The anesthesiologist will continue trying until successful* 2. *The operation will be cancelled as there are no other options* 3. *Pain medication in the drip* 4. *I do not know* |
|  |  |
| ***Procedural sedation and analgesia*** | |
| PSA1 | During sedation, as with general anaesthesia, the patient stops breathing spontaneously. |
|  | 1. *True* 2. *False* 3. *I do not know* |
| PSA2 | After sedation, patients can sometimes remember some occurrences from during the sedated period. |
|  | 1. *True* 2. *False* 3. *I do not know* |
| PSA3 | The patient must be fasted for a procedure performed under sedation. |
|  | 1. *True* 2. *False* 3. *I do not know* |
| PSA5 | What is true for both sedation and general anaesthesia? |
|  | 1. *The patient can’t remember anything about the procedure* 2. *The patient must be fasted* 3. *The patient is ventilated with a machine* 4. *All of the above* 5. *I do not know* |
| PSA6 | Are there procedures for which sedation is not enough and general anaesthesia is required? |
|  | 1. *Yes, there are* 2. *No, there are not* 3. *I do not know* |
