# Supplementary material for: Patient knowledge in anaesthesia: Psychometric development of the RAKQ–The Rotterdam anaesthesia Knowledge questionnaire
Source: PLoS One. 2024 Jul 12;19(7):e0299052. doi: 10.1371/journal.pone.0299052 (PMC11244777; doi:10.1371/journal.pone.0299052)
Supplement: Attachment — Submitted filename: PONE-D-23-17620_reviewed.pdf [file pone.0299052.s009.pdf]

PLOS ONE

Patient knowledge in anaesthesia: psychometric development of the RAKQ – the Rotterdam Anaesthesia Knowledge Questionnaire

--Manuscript Draft--

Manuscript Number:	PONE-D-23-17620
Article Type:	Research Article
Full Title:	Patient knowledge in anaesthesia: psychometric development of the RAKQ – the Rotterdam Anaesthesia Knowledge Questionnaire
Short Title:	Psychometric development of patient knowledge questionnaire on anaesthesia
Corresponding Author:	Sander van den van den Heuvel<br>Erasmus MC<br>Rotterdam, NETHERLANDS
Keywords:	Anaesthesia; Informed Consent; Surveys and Questionnaires; Psychometrics; Patient Education as Topic
Abstract:	The transition from in-person tot digital preoperative patient education requires effective methods for evaluating patients' understanding of the perioperative process, associated risks, and preoperative instructions to ensure informed consent.A validated knowledge questionnaire covering different anaesthesia techniques and preoperative instructions could fulfil this need.Therefore, we constructed a set of items covering knowledge on the most common anaesthesia techniques that require informed consent. Using a structured approach and Item Response Theory, we developed and psychometrically validated the Rotterdam Anaesthesia Knowledge Questionnaire (RAKQ). A team of anaesthetists and educational experts developed the initial set of 60 items,ensuring content and face validity. Next, based on exploratory factor analysis, we identifiedseven sub-questionnaires: General Anaesthesia – I (regardingwhat to expect), General Anaesthesia – II (regarding the risks),Spinal Anaesthesia, EpiduralAnaesthesia, Regional Anaesthesia, Procedural sedation and analgesia, and Generic Items.In total,50 multiple-choice items were selected based on factor loadings and clinical relevance, distributed over the seven sub-questionnaires.Through an iterative process to ensure that the sub-questionnaires met the criteria for Item Response Theory modelling, 40 items were selected.Finally, wedeveloped Item Response Theory models and evaluated their reliability.The newly developed RAKQ can be usedby practitioners to assess their patients' knowledge before consultation to better address knowledge gaps during consultation. Moreover, they can decide whether the level of knowledge is sufficient to obtain digital informed consent without face-to-face education.Researchers can use the RAKQ to compare new methods of patient education with traditional methods.
Order of Authors:	Sander van den van den Heuvel
	Hester van Eeren
	Sanne E. Hoeks
	Anna Panasewicz
	Philip Jonker
	Sohal Y. Ismail
	Jan J. van Busschbach
	Robert Jan Stolker
	Jan-Wiebe H. Korstanje
Additional Information:	
Question	Response
Financial Disclosure	This study was supported by a grant provided by the Dutch Ministry of Economic Affairs to JK (TKI grant No. EMCLSH200009). The sole responsibility for the content of

Powered by Editorial Manager® and ProduXion Manager® from Aries Systems Corporation

Enter a financial disclosure statement that describes the sources of funding for the work included in this submission. Review the submission guidelines for detailed requirements. View published research articles from *PLOS ONE* for specific examples.<br><br>This statement is required for submission and **will appear in the published article** if the submission is accepted. Please make sure it is accurate.<br><br>**Unfunded studies**<br>Enter: *The author(s) received no specific funding for this work.*<br><br>**Funded studies**<br>Enter a statement with the following details:<br>• Initials of the authors who received each award<br>• Grant numbers awarded to each author<br>• The full name of each funder<br>• URL of each funder website<br>• Did the sponsors or funders play any role in the study design, data collection and analysis, decision to publish, or preparation of the manuscript?<br>• **NO** - Include this sentence at the end of your statement: *The funders had no role in study design, data collection and analysis, decision to publish, or preparation of the manuscript.*<br>• **YES** - Specify the role(s) played.<br><br>* typeset	this publication lies with the authors. The funders had no role in study design, data collection and analysis, decision to publish, or preparation of the manuscript.
**Competing Interests**<br><br>Use the instructions below to enter a competing interest statement for this submission. On behalf of all authors, disclose any competing interests that could be perceived to bias this work—acknowledging all financial support and any other relevant financial or non-financial competing interests.<br><br>This statement is required for submission and **will appear in the published article** if the submission is accepted. Please make	I have read the journal's policy and the authors of this manuscript have the following competing interests: JK is an unpaid medical adviser for NovaCair B.V., a developer of digital preoperative screening software.

sure it is accurate and that any funding sources listed in your Funding Information later in the submission form are also declared in your Financial Disclosure statement.<br><br>View published research articles from *PLOS ONE* for specific examples.<br><br>**NO authors have competing interests**<br><br>Enter: *The authors have declared that no competing interests exist.*<br><br>**Authors with competing interests**<br><br>Enter competing interest details beginning with this statement:<br><br>*I have read the journal's policy and the authors of this manuscript have the following competing interests: [insert competing interests here]*<br><br>* typeset	
**Ethics Statement**<br><br>Enter an ethics statement for this submission. This statement is required if the study involved:<br><br>• Human participants<br>• Human specimens or tissue<br>• Vertebrate animals or cephalopods<br>• Vertebrate embryos or tissues<br>• Field research<br><br>Write "N/A" if the submission does not require an ethics statement.<br><br>General guidance is provided below. Consult the submission guidelines for detailed instructions. **Make sure that all information entered here is included in the Methods section of the manuscript.**	Ethical approval for this study was granted by the Medical Ethics Committee of Erasmus MC Rotterdam (MEC-2020-0468). Because there was no infringement on the physical and/or psychological integrity of the subject, the Medical Ethics Committee deemed the trial not to be subject to the Dutch Law on Medical Research. Written informed consent was obtained from all subjects. This study was conducted in compliance with the principles of the Declaration of Helsinki.

Powered by Editorial Manager® and ProduXion Manager® from Aries Systems Corporation

**Format for specific study types**<br><br>**Human Subject Research (involving human participants and/or tissue)**<br>• Give the name of the institutional review board or ethics committee that approved the study<br>• Include the approval number and/or a statement indicating approval of this research<br>• Indicate the form of consent obtained (written/oral) or the reason that consent was not obtained (e.g. the data were analyzed anonymously)<br><br>**Animal Research (involving vertebrate animals, embryos or tissues)**<br>• Provide the name of the Institutional Animal Care and Use Committee (IACUC) or other relevant ethics board that reviewed the study protocol, and indicate whether they approved this research or granted a formal waiver of ethical approval<br>• Include an approval number if one was obtained<br>• If the study involved *non-human primates*, add *additional details* about animal welfare and steps taken to ameliorate suffering<br>• If anesthesia, euthanasia, or any kind of animal sacrifice is part of the study, include briefly which substances and/or methods were applied<br><br>**Field Research**<br><br>Include the following details if this study involves the collection of plant, animal, or other materials from a natural setting:<br>• Field permit number<br>• Name of the institution or relevant body that granted permission	
**Data Availability**<br><br>Authors are required to make all data underlying the findings described fully available, without restriction, and from the time of publication. PLOS allows rare exceptions to address legal and ethical concerns. See the PLOS Data Policy and FAQ for detailed information.	Yes - all data are fully available without restriction

Powered by Editorial Manager® and ProduXion Manager® from Aries Systems Corporation

A Data Availability Statement describing where the data can be found is required at submission. Your answers to this question constitute the Data Availability Statement and **will be published in the article**, if accepted.<br><br>**Important:** Stating ‘data available on request from the author’ is not sufficient. If your data are only available upon request, select ‘No’ for the first question and explain your exceptional situation in the text box.<br><br>Do the authors confirm that all data underlying the findings described in their manuscript are fully available without restriction?	
**Describe where the data may be found in full sentences. If you are copying our sample text, replace any instances of XXX with the appropriate details.**<br><br>• If the data are **held or will be held in a public repository**, include URLs, accession numbers or DOIs. If this information will only be available after acceptance, indicate this by ticking the box below. For example: *All XXX files are available from the XXX database (accession number(s) XXX, XXX.*).<br>• If the data are all contained **within the manuscript and/or Supporting Information files**, enter the following: *All relevant data are within the manuscript and its Supporting Information files.*<br>• If neither of these applies but you are able to provide **details of access elsewhere**, with or without limitations, please do so. For example:<br><br>*Data cannot be shared publicly because of [XXX]. Data are available from the XXX Institutional Data Access / Ethics Committee (contact via XXX) for researchers who meet the criteria for access to confidential data.*<br><br>*The data underlying the results presented in the study are available from (include the name of the third party*	All source data and code files are available from the Dataverse database (https://dataverse.nl/). (DOI tba)

and contact information or URL).<br>• This text is appropriate if the data are owned by a third party and authors do not have permission to share the data.<br><br>* typeset	
Additional data availability information:	Tick here if the URLs/accession numbers/DOIs will be available only after acceptance of the manuscript for publication so that we can ensure their inclusion before publication.

Manuscript

Click here to access/download;Manuscript;Manuscript RAKQ - PLOS.docx

Patient knowledge in anaesthesia: psychometric development of the RAKQ – the Rotterdam Anaesthesia Knowledge Questionnaire

Short title: Psychometric development of patient knowledge questionnaire on anaesthesia

Sander F. van den Heuvel[1*], Hester van Eeren[2], Sanne E. Hoeks[1], Anna Panasewicz[3], Philip Jonker[1], Sohal Y. Ismail[2], Jan J. van Busschbach[2], Robert Jan Stolker[1], Jan-Wiebe H. Korstanje[1]

[1] Department of Anaesthesiology, Erasmus MC University Medical Centre Rotterdam, the Netherlands

[2] Department of Psychiatry, Erasmus MC University Medical Centre Rotterdam, the Netherlands

[3] Department of Anaesthesiology, Albert Schweitzer Hospital, Dordrecht, the Netherlands

* Corresponding author

Email: s.vandenheuvel@erasmusmc.nl

Abstract

must include summarized results from EFA & IRT

spelling

The transition from in-person tot digital preoperative patient education requires effective methods for evaluating patients' understanding of the perioperative process, associated risks, and preoperative instructions to ensure informed consent. A validated knowledge questionnaire covering different anaesthesia techniques and preoperative instructions could fulfil this need. Therefore, we constructed a set of items covering knowledge on the most common anaesthesia techniques that require informed consent. Using a structured approach and Item Response Theory, we developed and psychometrically validated the Rotterdam Anaesthesia Knowledge Questionnaire (RAKQ). A team of anaesthetists and educational experts developed the initial set of 60 items, ensuring content and face validity. Next, based on exploratory factor analysis, we identified seven sub-questionnaires: General Anaesthesia

sub-domains/facets/sections

– I (regarding what to expect), General Anaesthesia – II (regarding the risks), Spinal Anaesthesia, Epidural Anaesthesia, Regional Anaesthesia, Procedural sedation and analgesia, and Generic Items. In total, 50 multiple-choice items were selected based on factor loadings and clinical relevance, distributed over the seven sub-questionnaires. Through an iterative process to ensure that the sub-questionnaires met the criteria for Item Response Theory modelling, 40 items were selected. Finally, we developed Item Response Theory models and evaluated their reliability. The newly developed RAKQ can be used by practitioners to assess their patients' knowledge before consultation to better address knowledge gaps during

consultation. Moreover, they can decide whether the level of knowledge is sufficient to obtain digital informed consent without face-to-face education. Researchers can use the RAKQ to compare new methods of patient education with traditional methods.

Introduction

Informed consent is legally required for any medical procedure or therapy. Preoperative patient education on the procedure, risks, and necessary preparations are essential for obtaining informed consent for any type of anaesthesia (e.g. general, spinal, epidural, or regional anaesthesia). Therefore, it is an integral part of preoperative anaesthetic consultations. Today's technological advancements make digital preoperative education and screening feasible, with less direct involvement of the anaesthetist, less need for hospital real estate, and a lower burden on patients in terms of time and costs spent on travelling [1]. To facilitate the transition from face-to-face education and screening to digital education and screening, there is a need to compare novel teaching methods, for example educating patients using video animation, to a conventional face-to-face setting in terms of patients' knowledge level on anaesthesia. A psychometrically validated knowledge questionnaire covering all aspects of anaesthesia could be a tool to evaluate patients' knowledge before obtaining informed consent and a first step in digitally screening patients.

Although several questionnaires have been developed to measure knowledge on anaesthetic topics [2-5], only the questionnaire developed by Miller et al. was validated. However, it was intended for the parents of paediatric patients. Moreover, these earlier questionnaires covered only general anaesthesia and preoperative instructions. None of the questionnaires tested knowledge on other anaesthesia techniques. Furthermore, none of the questionnaires

were validated for use in different populations, and none were developed for use in daily practice to test individual patients' knowledge.

To assess patients' knowledge on anaesthesia effectively and validly in a digital setting, new psychometrically validated questionnaires are needed for a wider variety of anaesthesia types. Therefore, we designed this study to construct a set of items covering the most common anaesthesia techniques that require informed consent (i.e. general anaesthesia, spinal anaesthesia, epidural anaesthesia, regional anaesthesia, procedural sedation and analgesia (PSA), and generic items). The primary objectives were to (i) develop a set of items covering knowledge on different anaesthesia techniques, (ii) develop a scale along which this knowledge can be graded, and (iii) construct models using Item Response Theory (IRT) and determine the psychometric properties of the items.

Materials and methods

Ethical approval for this study was granted by the Medical Ethics Committee of Erasmus MC Rotterdam (MEC-2020-0468). Because there was no infringement on the physical and/or psychological integrity of the subject, the Medical Ethics Committee deemed the trial not to be subject to the Dutch Law on Medical Research. Written informed consent was obtained from all subjects. This study was conducted in compliance with the principles of the Declaration of Helsinki. ethical approval should be last paragraph in this section

Based on the methodology described by Boateng et al.[6] and Reeve et al.[7], we divided the process of developing a knowledge scale into three distinct phases: I) item development, II) scale development, and III) scale evaluation, as shown in Fig 1. This study aimed to create a comprehensive questionnaire covering six distinct knowledge domains regarding anaesthesia: general anaesthesia, spinal anaesthesia, epidural anaesthesia, regional anaesthesia, PSA, and generic items (e.g. preoperative instructions). The development and adaptation of the questionnaire are explained in the following steps, adhering to psychometric and clinical guidelines.

Fig 1. Steps in developing RAKQ (adapted from Boateng et al.[7] and Reeve et al.[1]). *Item Response Theory; †Differential item Functioning.

Phase I – Item development

Item development should go along with scale development
Theory should guide item development. Therefore, Phase I and II should be combined

1. Identification of domains and item generation

First, we conducted a literature search to collect sample questions from published questionnaires on patient knowledge regarding anaesthesia to guide item generation. Two anaesthetists then developed an item bank of 51 items covering the six aforementioned knowledge domains. The items were formulated as multiple-choice items with three to five answer categories, including an 'I do not know' option to discourage guessing. All the items had only one correct answer.

51 or 60 items? Different from Results

2. Content validity

To ensure content validity, all items were presented to 14 anaesthetists and three registrars in both a general hospital and an academic hospital. They were asked to accept or reject an item or suggest adaptation of an item. The resulting item bank was reviewed by an educational expert and psychologist, and the formulation of the items was adapted to ensure that language level B1 was not exceeded and to prevent ambiguous answer categories.

what is language level B1?

3. Face validity

To ensure face validity, this set of items was presented to three patients visiting the outpatient preoperative screening clinic. After completing the questionnaire, the patients were interviewed about the appropriateness of the questionnaire to measure their knowledge on anaesthesia, and comprehension of all items was assessed. Their comments were used to adapt the items.

Phase II – Scale development

1. Sampling and survey administration

This should be mentioned clearly as sample size determination for the analysis. In addition, this section is more suitable in Scale Evaluation phase.

As a rule of thumb, IRT analysis requires ten participants per item [8]. Based on the resulting number of items from the item development phase, at least 510 patients had to be included, which was met by including 577 patients visiting the preoperative outpatient clinic. Between September 2020 and November 2020, 319 patients were included in the Erasmus Medical Centre (Erasmus MC) in Rotterdam, a university hospital, and from April 2021 to May 2021, 258 patients were included in the Albert Schweitzer Hospital (ASZ) in Dordrecht, a general teaching hospital, both in the Netherlands. Patient inclusion criteria were a minimal age of 18 years, ability to read and understand Dutch, and planned elective surgery. Before administering the questionnaire in the ASZ, slight textual adjustments were made by a team of anaesthetists and a psychologist, based on remarks from participants in Erasmus MC. Care was taken not to change the content of the items or the answer categories. Special attention

was paid to the altered items during the scale-evaluation phase.

The questionnaires were sent by e-mail after the preoperative consultation had taken place. All participants completed all questionnaires, regardless of the anaesthesia technique they were educated on. The questionnaires were presented on a secure online survey platform. Age, sex and the planned anaesthesia technique were extracted from the hospital information system during the period of inclusion. This data was pseudonymised and the authors did not have access to information that could identify individual participants.

My personal view on this step, is that, this step was redundant, because the knowledge domains were decided earlier on during the development. In IRT context, each of this domain can be checked for the IRT parameters of interest and checked for unidimensionality. Oblique rotation is also not necessary, because each knowledge domain is not meant to be correlated and each knowledge domain is meant to stand alone from the start.

2. Extraction of factors

To explore latent factors within each predefined anaesthesia domain, we performed exploratory factor analysis using Multidimensional IRT (MIRT) with the R-package 'mirt' [9, 10]. To determine the number of latent factors that best described the data, we first fitted multiple MIRT models with an increasing number of factors using the Metropolis-Hastings Robbins-Monro algorithm [11]. We compared nested models using the Akaike information criterion (AIC), Bayesian Information Criteria (BIC), and chi-square difference tests. Although we took into account the BIC, in this exploratory phase we preferred the AIC over the BIC to decide which model to choose, because the AIC applies a smaller penalty than the BIC and thus reduces the risk of losing too much information.

Next, we explored the factor loadings in the optimal MIRT model option for each domain. To

Oblique rotation is not in line with unidimensionality assumption.

allow the factors to correlate, we used oblique rotation (Oblimin) [12]. The exploratory nature of the analysis and the large sample size made us consider items with factor loadings as low as 0.25 [13]. Guided by the factor loadings, we assembled item sets based on the following: factors needed to be conceptually interpretable, the first factor was prioritised above the next (since every next factor is extracted based on the residual of the previous factor), and a minimum of three items per factor was needed (to facilitate further analysis) [12]. If the item contributed conceptually to one of the factors, the cross-loading of an item on multiple factors was not used as a reason for removal. When an item did not load on the factor that was selected for the final item set, providing it was conceptually fitting that factor, it was deemed relevant from a clinical perspective and there were no doubts about the quality of the item, the item was kept in the item set during this phase of the scale development. The final selection of the factors was the set of scales evaluated in the next phase.

Phase III – Scale evaluation

1. Checking of assumptions IRT

Answer categories were dichotomised into correct vs. incorrect answers, where the category 'I don't know' was categorised as being incorrect. To fit an IRT model the assumptions of unidimensionality, local independence, and monotonicity must be met [7]. We assessed

these assumptions within the derived scale structure of the questionnaire based on the item and scale development. The assumptions were tested in an iterative process in which an assumption was tested again if the next assumption was not met for that scale.

A. Unidimensionality

Confirmatory factor analysis with weighted least square mean- and variance-adjusted estimator was performed to assess the unidimensionality of each scale using the R-package 'lavaan' [14]. Unidimensionality was accepted when the following criteria were met: a standardised root mean square residual (SRMR) value < 0.08, root mean square error of approximation (RMSEA) value < 0.06 and Scaled Comparative Fit Index (CFI) and Tucker-Lewis Index (TLI) values > 0.95 [15-20]. When unidimensionality was not satisfactory, items were chosen to be left out in subsequent analysis, based on factor loading and clinical relevance.

It is also recommended to check using unidimTest() function in ltm package which uses modified parallel analysis.

B. Monotonicity

We used the R-package 'mokken' [21, 22] to assess monotonicity and scalability and invariant item ordering (IIO) by fitting nonparametric IRT functions using Mokken scaling [23]. The assumption of monotonicity was considered met when no significant violations of

monotonicity were detected based on the z-test statistics. Scalability was assessed using the value of the scalability coefficient H of the entire scale and individual items (H_i). The H value of the entire scale was considered strong if H > 0.50, moderate if 0.40 ≤ H ≤ 0.50 and weak if 0.30 ≤ H ≤ 0.40 [21]. IIO was determined by assessing the number of significant violations of IIO per item based on the z-test statistic. When significant violations of monotonicity or IIO were detected or unscalable items were encountered, backward selection was applied by stepwise removal of the items with the highest violations or the lowest H coefficient until no violations were detected. This process ended when the H-coefficient of the entire scale was satisfactory [24].

C. Local independence

We used the R package 'lavaan' [14] to assess local independence. Local independence was met if the residual correlations between all the item pairs in the CFA model were < 0.20 [7]. When local dependencies were detected, we reviewed those items to determine the nature of the dependency and removed them when deemed necessary.

2. IRT model fitting

Once the assumptions were met, the item response functions, that is Rasch, 2PL, and 3PL

models, were fitted per scale, using the Expectation Maximization estimation algorithm within the 'mirt' package [10]. Nested models were compared using a likelihood ratio test, AIC, and BIC, and the best-fitting model was chosen [10].

The goodness of fit of the IRT models was assessed at item level using the S-X2 statistic for item fit, for which a p-value of <0.001 for an item was considered a misfit [7, 25, 26].

3. Differential Item Functioning

We evaluated Differential Item Functioning (DIF) with the R-package 'lordif' [27] using ordinal regression modelling with McFadden's pseudo $R^2 \geq 0.02$ being indicative of DIF (R^2_{12} suggests uniform DIF, R^2_{23} suggests non-uniform DIF) [28]. We evaluated age (two groups divided by the median age of the study sample), sex, the hospital the patient visited for preoperative screening (EMC vs. ASZ), level of education (tertiary vs. primary, secondary, and other), and whether the anaesthesia technique discussed during the preoperative consultation matched the tested knowledge domain of the scale (yes vs. no). To assess the magnitude and statistical relevance of DIF when an item was flagged for DIF, we compared the difference between the initial theta's (i.e. the estimated level of ability) and the theta's without items with DIF (i.e. purified theta's) on a scale level. This difference was then evaluated by plotting the differences due to DIF against the median standard error of measurement (SEm) [29]. Differences larger than the mean SEm were considered noticeable.

4. Information

In IRT, each response pattern results in a different theta and a different associated standard error. Therefore, the precision or reliability of an IRT model differs across the range of theta and is conceptualised as information. To investigate the information of the models, Test Information Curves were plotted together with the SE(theta), which was estimated using the Expected A Posteriori estimator, using the R-package ‘mirt’ [10]. When compared with classical testing, an SE(theta) of 0.32 or lower, corresponds to a reliability of 0.90 or higher, and can thus be considered a reliable measurement [30].

Statistics and data analysis

Software / packages used should be mentioned here instead of scattered at several places in the text.

Data were collected using LimeSurvey [31] and Gemstracker [32]. Data analysis was performed using R (version 4.2.0) [33]. Demographic and clinical characteristics were compared between the two hospitals and expressed as mean (SD) or number (percentage), where appropriate. Continuous variables were compared using Welch’s t-test, Mann-Whitney U test or Kruskal-Wallis test where appropriate, categorical variables were compared using the Chi-Square test.

Results

The results can be rearranged into Scale development (which includes item development) and Scale Evaluation.

In this study, we developed sets of items covering knowledge on different anaesthesia techniques and constructed scales in which this knowledge can be graded. We then constructed models using IRT and determined the psychometric properties of the items.

Phase I – Item development

This is too short to describe the item + scale development. Qualitative account of the development must be described and elaborated. The items should not be relegated to supplementary material.

Based on expert opinion, 60 items were developed within six predefined knowledge domains: Generic items, General anaesthesia, Spinal anaesthesia, Epidural anaesthesia, Regional anaesthesia and PSA. The full list of items, as a non-validated English translation of the Dutch original, that resulted after checking for content and face validity is shown in S1 Table.

Phase II – Scale development

To be combined with item development

1. Sampling and survey administration

More suitable in Scale Evaluation

Table 1 shows the demographic and clinical characteristics of the 577 participants who completed the questionnaires. The response rate was 55% in Erasmus MC and 28% in ASZ. Across all participants, the mean age was 60 (± 15) and 274 (48%) were female. The percentage of female participants was 41% versus 56% in Erasmus MC and ASZ, respectively (p=0.002). In ASZ, spinal and regional anaesthesia was planned more often compared to

Erasmus MC (20% versus 2% and 10% versus 3%, respectively, p<0.001). Other types of planned anaesthesia techniques, as well as age and highest level of education did not differ significantly between participants.

Table 1 Demographics and clinical characteristics of the two sample sites.

	**EMC***	**ASZ†**	**Total**	**P-value**
	n=319	*n=258*	*n=577*	
Age; y	59.3 (15.4)	60.1 (14.7)	59.7 (15.1)	0.910
Sex; female	130 (41)	144 (56)	274 (48)	0.002
Highest level of education				0.252
Primary	18 (6)	13 (5)	31 (5)	
Secondary	185 (58)	160 (64)	345 (61)	
Higher	102 (32)	78 (31)	180 (31)	
‘I don’t know’	3 (1)	0 (0)	3 (1)	
Other	11 (3)	0 (0)	11 (2)	
Anesthesia technique				
General anaesthesia	244 (77)	190 (74)	434 (75)	0.694
Spinal anaesthesia	6 (2)	52 (20)	58 (10)	<0.001
Epidural anaesthesia	22 (7)	14 (5)	36 (6)	0.701
Regional anaesthesia	9 (3)	25 (10)	34 (6)	0.002
Procedural sedation and analgesia	62 (20)	33 (13)	95 (17)	0.098

Values are mean (SD) or number of patients (valid percentage).

* Erasmus MC Rotterdam; †Albert Schweitzer Hospital Dordrecht

2. Extraction of factors

Again, I find this unnecessary for knowledge scale development.

Table 2 shows the factor loadings >0.25 for all items within each knowledge domain, based on the optimal number of factors as determined by model comparison (S2 Table). Items

retained in the final item set(s) are printed in bold. The domains Regional anaesthesia, Spinal anaesthesia, Epidural anaesthesia and PSA all resulted in two factors, but were reduced to a single set of items per domain, including several items that did not load on the first factor, because of their clinical and conceptual relevance. The Generic items domain consisted of three factors and was reduced to a single set of items, because factors 2 and 3 consisted of too few items after allocating cross-loading items to factor 1. The General anaesthesia domain was reduced to two sets of items. Conceptually, factor 1 consisted of items relating to direct perioperative care ('General anaesthesia – I'), and factor 4 comprised of items concerning complications or side-effects ('General anaesthesia – II'). Factor 2 within General anaesthesia consisted of too few items when cross-loading was taken into account, and factor 3 was conceptually not interpretable. Items from factors 2 and 3 that fitted the construct of factor 4 were included in the 'General anaesthesia – II' item set for further analysis.

Table 2 Factor loadings for exploratory factor analysis.

Generic items	**Factor 1**	Factor 2	Factor 3	*Regional anaesthesia*	**Factor 1**	Factor 2
**GEN11**	**0.707**		0.439	**RA6**	**0.822**	
**GEN10**	**0.581**		-0.311	**RA7**	**0.457**	
**GEN3**	**0.543**	0.266		**RA2**	**0.444**	0.253
**GEN8**	**0.483**			**RA3**	**0.384**	0.417
**GEN4**	**0.345**			**RA5**	**0.383**	
**GEN12**	**0.252**			**RA4**	*	0.827
**GEN2**	*			RA1		0.267

GEN5		0.760	
GEN1		0.665	
GEN7			0.905
GEN6			
GEN9			

General anaesthesia	**Factor 1**	Factor 2	Factor 3	**Factor 4**
**GA9**	**0.890**			
**GA3**	**0.647**			
**GA2**	**0.597**		0.263	
**GA7**	**0.530**	0.302		
**GA10**	0.528			**0.392**
**GA5**	**0.430**			0.402
**GA1**	**0.411**			0.288
**GA11**	**0.387**			
**GA12**	0.292		0.547	*
**GA14**	0.263		0.283	**0.442**
**GA4**	**0.262**			
**GA13**		0.867		*
**GA15**		0.637	0.292	*
GA8			0.492	
**GA6**				**0.814**
**GA16**				**0.268**

Spinal anaesthesia	**Factor 1**	Factor 2
**SA6**	**0.938**	
**SA7**	**0.757**	
**SA12**	**0.752**	
**SA9**	**0.735**	
**SA13**	**0.661**	
**SA10**	**0.626**	
**SA4**	**0.620**	
**SA5**	**0.488**	0.327
**SA3**	**0.443**	
**SA2**	**0.428**	0.336
**SA11**	**0.355**	0.312
SA8	0.286	0.577
**SA1**	*	0.944

Epidural	**Factor 1**	Factor 2
**EA6**	**0.795**	
**EA4**	**0.771**	
**EA5**	**0.468**	
**EA3**	**0.423**	0.339
**EA1**	*	0.747
EA2		0.870

Procedural sedation and analgesia	**Factor 1**	Factor 2
**PSA2**	**0.765**	
**PSA1**	**0.638**	
**PSA6**	**0.616**	
**PSA5**	**0.447**	
**PSA3**	*	0.802
PSA4		0.522

Bold items were included for further analyses. Items with an asterisk (*) did not load on the factor, but were included based on conceptual fit to another factor.

My general comment on this, is that scales should also rely on theoretical and qualitative aspects. These sub-questionnaires were determined by EFA, which I find, from the way it was performed, guided by theoretical and qualitative judgements. I addition, EFA was performed with oblique (correlation) rotation, where orthogonal rotation (no correlation) is a better option if the aim is to look for unidimentional scales. There was no mention of estimation method used for EFA, and where the estimation method is suitable for binary response option.

Phase III – Scale evaluation

1. Checking of assumptions IRT

The assessment of IRT assumptions and consecutive adjustments of the item sets is an iterative process which is elaborated on below. The final assessment of all IRT assumptions is presented in Tables 3a, 3b, and 3c.

Table 3a Final assessment of Unidimensionality before IRT modelling can be undertaken.

Sub-questionnaire	Unidimensionality			
	SRMR*	RMSEA†	Scaled CFI‡	Scaled TLI¶
Generic items	0.015	0.000	1	1.034
General anaesthesia - I	0.072	0.029	0.993	0.988
General anaesthesia - II	0.066	0.038	0.973	0.956
Spinal anaesthesia	0.061	0.045	0.982	0.977
Regional anaesthesia	0.018	0.000	1.000	1.027
Epidural anaesthesia	0.019	0.000	1	1.014
Procedural sedation and analgesia	0.050	0.044	0.977	0.954

* Standardized Root Mean Squared Residual; † Root Mean Square Error of Approximation; ‡ Comparative Fit Index; ¶ Tucker-Lewis Index

Table 3b Final assessment of Monotonicity before IRT modelling can be undertaken.

Sub-questionnaire	Monotonicity			
	Scalability coefficients H	Scalability coefficients H_i	Violations monotonicity	Violations IIO*
Generic items	0.307	0.27 - 0.35	none	none
General anaesthesia - I	0.441	0.32 - 0.47	none	none
General anaesthesia - II	0.329	0.30 - 0.43	none	none
Spinal anaesthesia	0.439	0.37 - 0.61	2, none critical	none
Regional anaesthesia	0.349	0.28 - 0.45	none	none
Epidural anaesthesia	0.391	0.28 - 0.44	none	none
Procedural sedation and analgesia	0.316	0.22 - 0.36	none	none

Violations with a crit value >40 were deemed critical.

* Invariant Item Ordering

Table 3c Final assessment of Local Independence before IRT modelling can be undertaken.

Sub-questionnaire	Local Independence
	Residual correlation
Generic items	none
General anaesthesia - I	GA4 & GA5 (0.267)
General anaesthesia - II	none
Spinal anaesthesia	none
Regional anaesthesia	none
Epidural anaesthesia	none
Procedural sedation and analgesia	none

Generic items The initial set of seven items met the unidimensionality criteria, but was not scalable (H=0.202; all items H_i<0.3). To improve scalability, the items GEN10, GEN4, and GEN12 were removed. The reduced set of four items met the unidimensionality criteria and was evaluated as weakly scalable (H=0.307; two items H_i<0.3). No serious violations of monotonicity, violations of IIO or local dependencies were detected.

General anaesthesia – I The initial set of eight items met the unidimensionality criteria and was weakly scalable (H=0.329; two items H_i<0.3). Analysis of IIO revealed that after removing two items (GA1 and GA11), no violation of IIO was detected. The reduced set of six items met the unidimensionality criteria and was moderately scalable (H=0.441; no item H_i <0.3).

No serious violations of monotonicity were detected. Two items showed local dependence (GA4 and GA5), but apart from being very easy questions, no clinically relevant correlation could be found, and they were preserved in the item set.

General anaesthesia – II The initial set of seven items did not meet the unidimensionality criteria (SRMR=0.086). After reviewing the three items that did not load on the latent factor in Phase II and removing the clinically least relevant item (GA13), the reduced set of six items met the unidimensionality criteria. The item-set was weakly scalable (H=0.329; no item H_i<0.3). No serious violations of monotonicity, violations of IIO or local dependencies were detected.

Spinal anaesthesia The initial set of 12 items met the unidimensionality criteria and was moderately scalable (H=0.410; one item H_i<0.3). Analysis of IIO revealed that after removing two items (SA3 and SA10), no violation of IIO was detected. The reduced set of ten items met the unidimensionality criteria and was moderately scalable (H=0.439; no item H_i<0.3). No serious violations of monotonicity or local dependencies were detected.

Regional anaesthesia The initial set of six items met all but one unidimensionality criterion (scaled TLI) but was not scalable (H=0.252; four items H_i<0.3). To improve scalability, items

RA4 and RA5 were removed. The reduced set of four items met the unidimensionality criteria and was weakly scalable (H=0.349; one item H_i <0.3). No serious violations of monotonicity, violations of IIO or local dependencies were detected.

Epidural anaesthesia The initial set of five items met the unidimensionality criteria and was weakly scalable (H=0.391; one item H_i <0.3). No serious violations of monotonicity, violations of IIO or local dependencies were detected.

Procedural sedation and analgesia The initial set of five items met the unidimensionality criteria and was weakly scalable (H=0.316; four items H_i <0.3). No serious violations of monotonicity, violations of IIO or local dependencies were detected.

2. IRT model fitting

I find the decision to test out and mix different IRT models difficult to justify here. The decision must be based on why you want to use such model (ie to determine difficulty only assuming same discrimination etc), not because the model fit the data.

S3 Table shows comparisons between the Rasch, 2PL, and 3PL models for each scale. The Rasch model was the best fitting model for the Generic items, General anaesthesia – II and Regional anaesthesia scales. The 2PL-model was the best fitting model in the General anaesthesia – I, Spinal anaesthesia, Epidural anaesthesia, and PSA domains. Table 4 shows the item parameters per scale and S4 Figure shows the item characteristic curves (ICC) per scale. The item parameters and fit are listed in Table 4. No item misfit was detected.

Table 4 IRT-model item parameters and Item Fit (p-values)

Item ID	IRT-model item parameters		Item fit
	Difficulty	Discrimination	p(S-χ^2)
Generic items			
GEN2	0.224	1	0.035
GEN3	-1.378	1	0.143
GEN8	-2.201	1	0.406
GEN11	-1.984	1	0.210
General anaesthesia - I			
GA2	-0.743	2.075	0.533
GA3	-1.594	1.822	0.208
GA4	-3.232	1.072	0.658
GA5	-2.913	1.694	0.732
GA7	-0.925	2.025	0.759
GA9	-0.677	2.849	0.588
General anaesthesia - II			
GA6	2.517	1	0.079
GA10	-0.770	1	0.105
GA14	-2.487	1	0.665
GA16	-1.365	1	0.605
GA12	-1.592	1	0.142
GA15	0.244	1	0.105
Spinal anaesthesia			
SA1	0.212	1.444	0.467
SA2	0.862	1.499	0.137
SA4	0.812	1.838	0.431
SA5	0.606	1.881	0.036
SA6	0.408	2.408	0.173
SA7	-0.729	2.209	0.075
SA9	0.023	2.007	0.119
SA11	1.681	1.146	0.287
SA12	-0.700	1.438	0.125
SA13	-0.062	1.649	0.549
Regional anaesthesia			
RA2	1.833	1	0.595
RA3	1.674	1	0.543

RA6	0.476	1	0.149
RA7	-1.246	1	0.115
Epidural anaesthesia			
EA1	1.623	1.537	0.209
EA3	-0.069	1.521	0.124
EA4	0.287	1.908	0.331
EA5	1.488	0.858	0.193
EA6	0.383	2.380	0.407
Procedural sedation and analgesia			
PSA1	-0.683	1.835	0.560
PSA2	0.536	1.367	0.157
PSA3	-1.657	0.733	0.748
PSA5	0.566	1.195	0.058
PSA6	-1.470	1.277	0.546

3. Differential Item Functioning

Table 5 shows which items have DIF and the magnitude and type of DIF (only McFadden's pseudo $R^2 \geq 0.02$ is shown). Overall, no items were flagged for DIF regarding sex, seven items were flagged for DIF regarding age, one item was flagged for DIF regarding anaesthesia technique, four items were flagged for DIF regarding the sample hospital, and four items were flagged for DIF regarding the level of education. The impact of DIF on the total scores for each scale was well below the mean SEm, and its impact was negligible. Of the items that were slightly modified between the hospitals (GA2 and GA3), only GA2 was flagged for DIF regarding the sample hospital, but its impact was negligible. A more detailed exploration of DIF within the scales is shown in online Supporting Information Document S4.

Table 5 Differential Item Functioning (pseudo-R^2) per model.

Item ID	Differential Item Functioning									
	Sex		Age		Technique		Hospital		Education	
	R^2_{12}	R^2_{23}	R^2_{12}	R^2_{23}	R^2_{12}	R^2_{23}	R^2_{12}	R^2_{23}	R^2_{12}	R^2_{23}
Generic items										
GEN3				0.106						
GEN11				0.141						
General anaesthesia - I										
GA2			0.034							
GA4			0.022	0.030					0.030	0.028
GA5			0.076							
General anaesthesia - II										
GA6										0.073
GA10							0.124			
GA14										0.064
GA16								0.040		
GA12								0.032		
Spinal anaesthesia										
SA11			0.055							
Regional anaesthesia										
RA2			0.037							
Epidural anaesthesia										
EA1					0.063					

Only items with Differential Item Functioning are mentioned. All other items had no Differential item Functioning.

4. Information

The Test Information Curves and SE(theta) for each scale are shown in Fig 2. The order of the scales regarding difficulty (i.e. the theta's at which the scale is most informative) was from most difficult to least difficult: Regional anaesthesia, Epidural anaesthesia, Spinal anaesthesia, PSA, General anaesthesia I, General anaesthesia II, and Generic items.

Fig 2. Test Information (I) and Standard Errors (SE) Curves of the seven scales.

Final questionnaires

S5 Table shows the English translation of the seven final questionnaires on preoperative knowledge on anaesthesia techniques after scale development and evaluation.

Discussion

This is the first elaborative effort to develop a validated questionnaire that covers the main domains of patient knowledge in anaesthesia in adult care and to evaluate its psychometric properties. This questionnaire, the RAKQ, contains a set of seven questionnaires covering five different anaesthesia techniques and one generic domain, with a total of 40 multiple-choice items. Through a psychometric evaluation, we created unidimensional questionnaires and provided an IRT model for each domain.

Several questionnaires have been developed to measure knowledge on anaesthetic topics [2-5] in the past. However, the RAKQ is the first questionnaire to cover all clinically relevant anaesthesia techniques, such as spinal, epidural, and regional anaesthesia, and PSA. Furthermore, the RAKQ has undergone extensive psychometric evaluation, using IRT for the first time in a preoperative knowledge questionnaire. Trustworthy alternatives to in-person patient education on all topics of anaesthesia can only be developed using a validated instrument, such as the RAKQ.

Methodological considerations

A deductive approach was used to ensure content and face validity in the item development phase (Phase I). This implies that the generation of items was based on predefined domains

formulated by experts. However, during exploratory factor analysis, some items deemed important had low factor loadings (<|0.4|). This could mean that different underlying constructs can be identified, hindering our efforts to create unidimensional scales within predefined knowledge domains. Nevertheless, based on discussions within the team and the fact that the data was collected from a large multicentre sample [12], we proceeded with a lower threshold (>|0.25|). Subsequently, the resulting lists of items were evaluated using confirmatory factor analysis, which confirmed the unidimensionality of the individual sub-questionnaires. The resulting sub-questionnaires can inform the clinician about patients' knowledge on these domains, while also helping to shape and refine the constructs.

Scalability is also important during the development of a valid scale using IRT [7]. We found weak to moderate scalability for all scales, indicating how difficult it is to scale knowledge on relatively broad subjects, such as knowledge on anaesthesia. Improvements in scalability could result in more items concerning less broad subjects. This would inevitably lead to lengthier questionnaires that are less user-friendly. Still, we believe that improving scalability would be the first step in accurately measuring patients' knowledge, since we aim for tailored consultation and education based on the questionnaire.

Future directions

The RAKQ can be used to assess the level of knowledge of patients regarding the perioperative process, risks, and preoperative preparations in both clinical practice and research settings. When applied after digital education and before consultation, consulting anaesthetists can pay particular attention to gaps in knowledge. Moreover, patients can automatically be offered additional information even before preoperative consultation. Furthermore, when a fully digital preoperative screening is considered for selected (low-risk) patients, the RAKQ can indicate whether patients have truly understood the information or whether additional education is needed for a well-informed consent. The RAKQ can be used as a research tool to compare new methods of patient education with traditional methods. The practical application of the RAKQ and its acceptance by patients and anaesthetists as a tool to optimise preoperative education should be evaluated in clinical settings.

As a next step, computerised-adaptive test (CAT) can be developed with the difficulty and discriminatory parameters provided by IRT modelling. With a sufficiently large item pool with items that are sufficiently discriminatory and differ across their difficulty levels, CAT can be used to reliably measure the level of knowledge with a minimal, individualised subset of items to reduce the burden on patients. As can be deduced from the Information Curves, our questionnaires differ in their overall difficulty, and the item sets require further diversification regarding the difficulty of the items to facilitate meaningful CAT. Further

research should focus on diversifying the level of difficulty such that discrimination between an acceptable and unacceptable level of knowledge is possible.

In summary, the set of questionnaires presented in this paper is the first to cover multiple commonly used anaesthesia techniques and the first to be psychometrically validated using IRT. We believe that these questionnaires are a solid foundation on which to further develop knowledge scales and explore computerised-adaptive testing. This can pave the way for trustworthy digital informed consent, which could reduce patient burden and optimise the efficiency of preoperative care.

Acknowledgments

The authors would like to thank Dr. M. Vereen and Dr. E. Galvin for the English translation of the RAKQ.

References

1. Kamdar NV, Huverserian A, Jalilian L, Thi W, Duval V, Beck L, et al. Development, Implementation, and Evaluation of a Telemedicine Preoperative Evaluation Initiative at a Major Academic Medical Center. Anesthesia and Analgesia. 2020;131(6):1647-56. doi: 10.1213/ANE.0000000000005208. PubMed PMID: 32841990.

2. Kakinuma A, Nagatani H, Otake H, Mizuno J, Nakata Y. The effects of short interactive animation video information on preanesthetic anxiety, knowledge, and interview time: a randomized controlled trial. Anesth Analg. 2011;112(6):1314-8. Epub 2011/02/25. doi: 10.1213/ANE.0b013e31820f8c18. PubMed PMID: 21346166.

3. Miller KM, Wysocki T, Cassady JF, Cancel D, Izenberg N. Validation of Measures of Parents' Preoperative Anxiety and Anesthesia Knowledge. Anesthesia & Analgesia. 1999;88(2):251-7. doi: 10.1213/00000539-199902000-00005. PubMed PMID: 00000539-199902000-00005.

4. Snyder-Ramos SA, Seintsch H, Bottiger BW, Motsch J, Martin E, Bauer M. [Development of a questionnaire to assess the quality of the preanesthetic visit]. Anaesthesist. 2003;52(9):818-29. Epub 2003/09/25. doi: 10.1007/s00101-003-0551-6. PubMed PMID: 14504809.

5. Zvara DA, Mathes DD, Brooker RF, McKinley AC. Video as a patient teaching tool: does it add to the preoperative anesthetic visit? Anesth Analg. 1996;82(5):1065-8.

Epub 1996/05/01. doi: 10.1097/00000539-199605000-00033. PubMed PMID: 8610869.

6. Boateng GO, Neilands TB, Frongillo EA, Melgar-Quinonez HR, Young SL. Best Practices for Developing and Validating Scales for Health, Social, and Behavioral Research: A Primer. Front Public Health. 2018;6:149. Epub 2018/06/27. doi: 10.3389/fpubh.2018.00149. PubMed PMID: 29942800; PubMed Central PMCID: PMC6004510.

7. Reeve BB, Hays RD, Bjorner JB, Cook KF, Crane PK, Teresi JA, et al. Psychometric evaluation and calibration of health-related quality of life item banks: plans for the Patient-Reported Outcomes Measurement Information System (PROMIS). Med Care. 2007;45(5 Suppl 1):S22-31. Epub 2007/04/20. doi: 10.1097/01.mlr.0000250483.85507.04. PubMed PMID: 17443115.

8. Nunnally JC. Psychometric theory. 2nd ed. ed. New York ;: McGraw-Hill; 1978.

9. Immekus JC, Snyder KE, Ralston PA. Multidimensional Item Response Theory for Factor Structure Assessment in Educational Psychology Research. Frontiers in Education. 2019;4. doi: 10.3389/feduc.2019.00045.

10. Chalmers RP. mirt: A Multidimensional Item Response Theory Package for the R Environment. Journal of Statistical Software. 2012;048(i06).

11. Garnier-Villarreal M, Merkle EC, Magnus BE. Between-Item Multidimensional

IRT: How Far Can the Estimation Methods Go? Psych. 2021;3(3):404-21. doi: 10.3390/psych3030029.

12. Worthington RL, Whittaker TA. Scale Development Research: A Content Analysis and Recommendations for Best Practices. The Counseling Psychologist. 2006;34(6):806-38. doi: 10.1177/0011000006288127.

13. Keenan AP, James PS. Applied Multivariate Statistics for the Social Sciences : Analyses with SAS and IBM's SPSS, Sixth Edition. New York: Routledge; 2016.

14. Rosseel Y. lavaan: An R Package for Structural Equation Modeling. Journal of Statistical Software. 2012;48(2):1 - 36. doi: 10.18637/jss.v048.i02.

15. MacDonald RP. Test theory : a unified treatment. Mahwah [etc.]: Lawrence Erlbaum Associates; 1999.

16. Kline RB. Principles and practice of structural equation modeling. New York: Guilford Press; 2016.

17. Bentler PM. Comparative fit indexes in structural models. Psychol Bull. 1990;107(2):238-46. PubMed PMID: 2320703.

18. West SG, Finch JF, Curran PJ. SEM with nonnormal variables. In: Hoyle RH, editor. Structural equation modeling : concepts, issues, and applications. Thousand Oaks, Calif.: Sage; 1995. p. 56-75.

19. Hu Lt, Bentler PM. Cutoff criteria for fit indexes in covariance structure analysis:

Conventional criteria versus new alternatives. Structural Equation Modeling: A Multidisciplinary Journal. 1999;6(1):1-55. doi: 10.1080/10705519909540118.

20. Browne MW, Cudeck R. Alternative ways of assessing model fit. In: Bollen KA, Long JS, editors. Testing structural equation models. Newbury Park, CA: Sage Publications; 1993.

21. van der Ark LA. Mokken Scale Analysis in R. Journal of Statistical Software. 2007;20(11):1 - 19. doi: 10.18637/jss.v020.i11.

22. van der Ark A. New Developments in Mokken Scale Analysis in R. J Stat Softw. 2012;48. doi: 10.18637/jss.v048.i05.

23. Wind S. Examining the Psychometric Quality of Multiple-Choice Assessment Items using Mokken Scale Analysis. Journal of applied measurement. 2016;17:142-65.

24. van Schuur W. Ordinal Item Response Theory: Mokken Scale Analysis. Thousand Oaks, California2011.

25. Luijten MAJ, Van Litsenburg RRL, Terwee CB, Grootenhuis MA, Haverman L. Psychometric properties of the Patient-Reported Outcomes Measurement Information System (PROMIS®) pediatric item bank peer relationships in the Dutch general population. Quality of Life Research. 2021;30(7):2061-70. doi: 10.1007/s11136-021-02781-w.

26. Orlando M, Thissen D. Further investigation of the performance of S-X²: An item fit index for use with dichotomous item response theory models. Applied Psychological Measurement. 2003;27(4):289-98. doi: 10.1177/0146621603027004004.

27. Choi SW, Gibbons LE, Crane PK. lordif: An R Package for Detecting Differential Item Functioning Using Iterative Hybrid Ordinal Logistic Regression/Item Response Theory and Monte Carlo Simulations. J Stat Softw. 2011;39(8):1-30. Epub 2011/05/17. doi: 10.18637/jss.v039.i08. PubMed PMID: 21572908; PubMed Central PMCID: PMCPMC3093114.

28. Crins MHP, Terwee CB, Ogreden O, Schuller W, Dekker P, Flens G, et al. Differential item functioning of the PROMIS physical function, pain interference, and pain behavior item banks across patients with different musculoskeletal disorders and persons from the general population. Quality of Life Research. 2019;28(5):1231-43. doi: 10.1007/s11136-018-2087-x.

29. Kleinman M, Teresi JA. Differential item functioning magnitude and impact measures from item response theory models. Psychol Test Assess Model. 2016;58(1):79-98. Epub 2016/01/01. PubMed PMID: 28706769; PubMed Central PMCID: PMCPMC5505278.

30. Klaufus LH, Luijten MAJ, Verlinden E, Van Der Wal MF, Haverman L, Cuijpers P, et

al. Psychometric properties of the Dutch-Flemish PROMIS® pediatric item banks Anxiety and Depressive Symptoms in a general population. Quality of Life Research. 2021;30(9):2683-95. doi: 10.1007/s11136-021-02852-y.

31. Limesurvey GmbH. LimeSurvey: An Open Source survey tool. Hamburg, Germany2020.

32. Erasmus MC and Equipe Zorgbedrijven. GemsTracker ©. 1.9.0 ed2011.

33. R Core Team. R: A language and environment for statistical computing. R Foundation for Statistical Computing. Vienna, Austria2022.

Supporting Information captions

S1 Table: List of items resulting from Phase I – Item development.

S2 Table: Comparison of nested multidimensional Item Response Theory models for exploratory factor analysis.

S3 Table: Comparison of 1-, 2- and 3-PL Item Response Theory models.

S4 Figure: Item Response Functions per scale.

S5 Table: Final questionnaires

S6 Document: Differential Item Functioning

S7 Table: Glossary of terms

 Click here to access/download;Figure;Fig1.tif

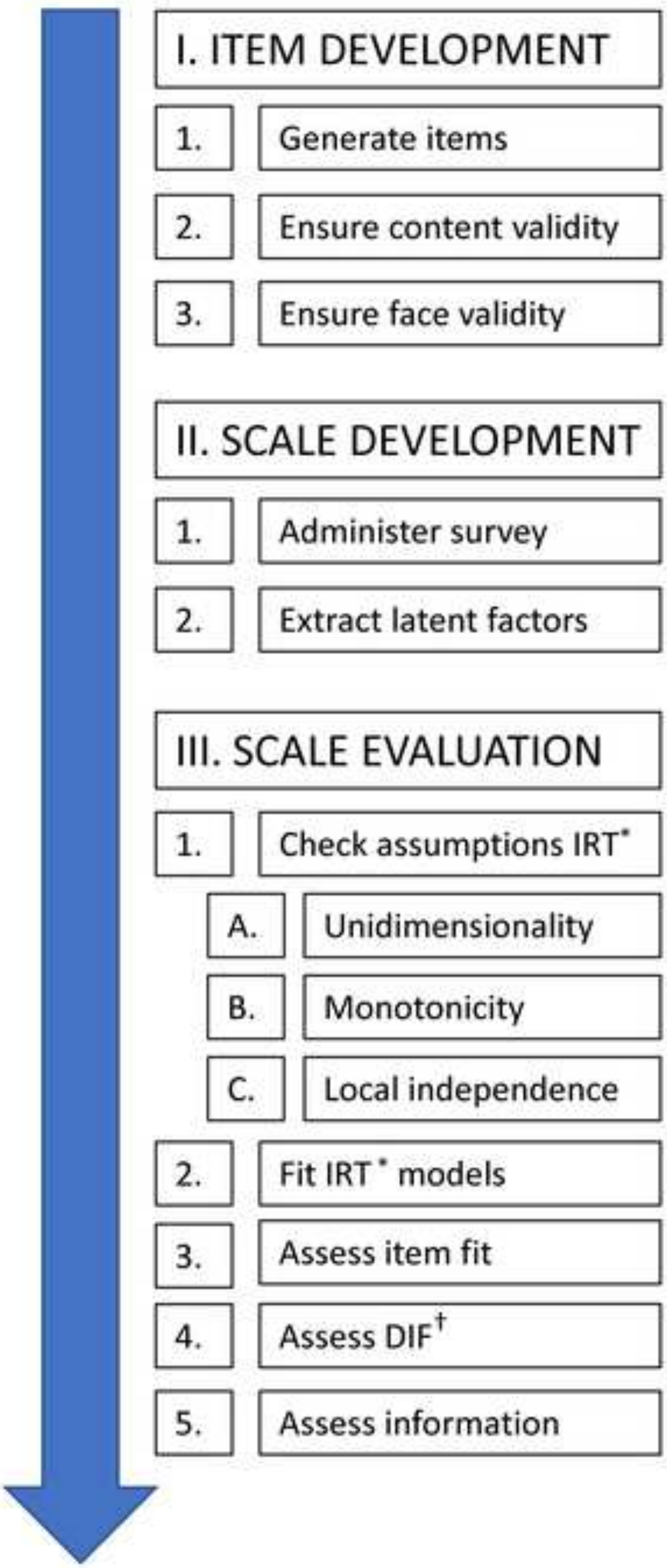

Figure 2 Click here to access/download;Figure;Fig2.tif

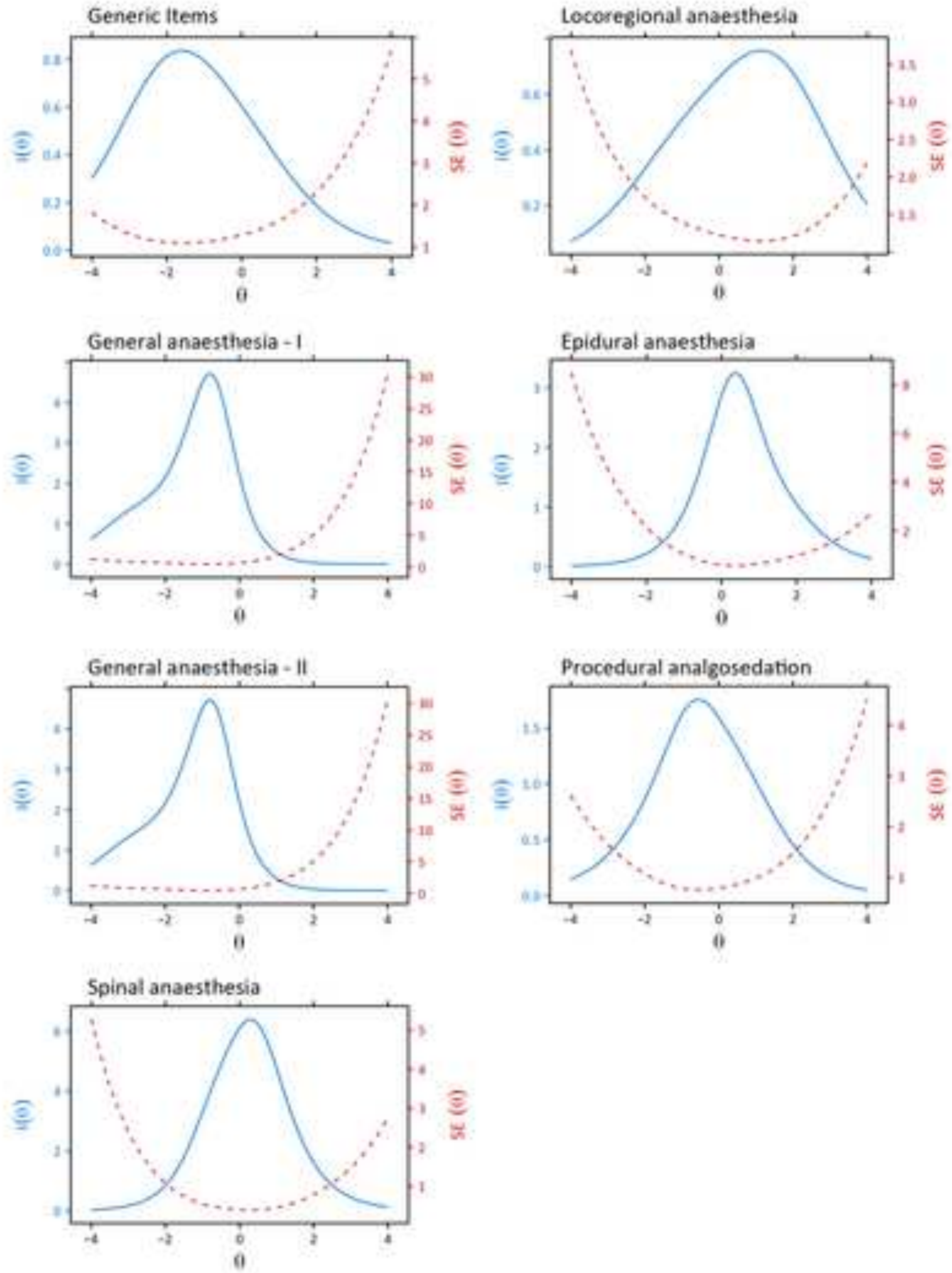

S1 Table

Click here to access/download
Supporting Information
S1 Table.docx

S2 Table

Click here to access/download
Supporting Information
S2 Table.docx

S3 Table

Click here to access/download
Supporting Information
S3 Table.docx

S4 Figure

Click here to access/download
Supporting Information
S4 Figure.tif

S5 Table

Click here to access/download
Supporting Information
S5 Table.docx

S7 Table

Click here to access/download
Supporting Information
S7 Table.docx

Click here to access/download
Supporting Information
S6 Document.docx
