# Supplementary material for: Patient knowledge in anaesthesia: Psychometric development of the RAKQ–The Rotterdam anaesthesia Knowledge questionnaire
Source: PLoS One. 2024 Jul 12;19(7):e0299052. doi: 10.1371/journal.pone.0299052 (PMC11244777; doi:10.1371/journal.pone.0299052)
Supplement: Attachment — Submitted filename: Response to the reviewers.docx [file pone.0299052.s010.docx]

# Response to the reviewers

## Reviewer #1:

1. **Remark**: The study can be better described by Phase 1 - Scale development and Phase 2 - Scale evaluation/validation. Scale and item development must be developed together, not separately.

**Answer**: We agree with your observation that the development of items and scales must be carried out concurrently, where we may have given the impression that the phases are separate. To accurately convey the message that item and scale development are interconnected, we have modified the headings and Figure 1 as you proposed.

2. **Remark**: EFA was used in this study to determine suitable latent dimensions from the data. In my opinion, for knowledge scale, this is redundant. If the items were developed while keeping in mind the scale / concepts they are supposed to represent, unidimensionality will be quite obvious and any items that do not fit in IRT model can be removed from the scale during IRT.
   For the IRT analysis, the sub-questionnaires should be decided based on qualitative and theoretical standpoint, not by EFA findings.

**Answer**: We appreciate reviewer's insight regarding the implementation of Exploratory Factor Analysis (EFA) in our study to identify latent dimensions, particularly in relation to the knowledge scales. While we acknowledge the validity of this perspective, and on which we agree in other circumstances, we would like to explain our rationale for employing EFA in the current study. Our questionnaire was developed from the perspective of an anaesthetist, keeping in mind the patient perspective, but the questions where not developed solely from the patient's perspective. Although we had a notion of the various domains within our questionnaire from the anaesthetist standpoint, we were uncertain whether they truly aligned with patients' perceptions. Through our analysis, we discovered that unidimensionality was not completely evident, especially for the overall anaesthesia set, which, when divided into multiple questionnaires, in our opinion, would serve as a more robust foundation for subsequent Item Response Theory (IRT) analysis, in which it is approached to take into account the patient perspective more accurately.

We added an extra sentence to the third paragraph in the discussion:

“This implies that the generation of items was based on predefined domains formulated by experts. Since this was a first endeavour in constructing a comprehensive knowledge questionnaire on anaesthesia, we conducted exploratory factor analysis to explore underlying latent constructs which could have been less obvious at forehand. During exploratory factor analysis we found some items deemed important had low factor loadings (<|0.4|). This could mean that indeed different underlying constructs can be identified, hindering our efforts to create unidimensional scales within predefined knowledge domains. Nevertheless, based on discussions within the team and the fact that the data was collected from a large multicentre sample [11], we proceeded with a lower threshold (>|0.25|). In future studies, it could be valuable to also include the patient perspective in developing items, which could enhance unidimensionality of the scales.”

(line 422-432)

3. **Remark**: I find the decision to test out and mix different IRT models difficult to justify in this study. The decision must be based on why you want to use such model (ie to determine
    difficulty only assuming same discrimination etc), not because the model fit the data.

**Answer**: We recognize that in developing a scale based on supposed concepts, those scales should represent the concepts and unidimensionality should be obvious. Furthermore, we subscribe to the remark that when implementing a questionnaire, e.g. when designing a Computerized Adaptive Test (CAT), a theoretical rationale for the choice of the model should be formulated. The 2-PL model provides more opportunities to differentiate between patients with sufficient or insufficient knowledge, with the contribution of the items to the estimation of the knowledge level being different. An argument for using a 1-PL model is the premise that every question is of equal importance in estimating the knowledge level. Given the exploratory nature of this study, we sought to provide information on the quality of the items and different models. When the development of a CAT is undertaken, a choice should be made based on the characteristics of the data as well as on the rationale behind the choice for a 1- or 2-PL model. To convey this message in the manuscript we added the following passage to the discussion:

“Given the exploratory nature of our study, we compared the relative fit of 1-, 2- and 3-PL models to assess which model showed the best fit to the data. The 3-PL models never showed to be an enhancement over the 2-PL models, in line with our approach to reduce the element of guessing by adding an additional response option, “ I don’t know”. It should be noted that when employing the questionnaire, a choice must be made between a 1-PL and 2-PL model. This decision should not only be based on the fit of the model to the data, but also on more fundamental considerations. A 2-PL model provides more opportunities to differentiate between patients with sufficient or insufficient knowledge, with the contribution of the items to the estimation of the knowledge level being different. Conversely, an argument for using a 1-PL model is the premise that every question is of equal importance in estimating the knowledge level.” (lines 448-455)

Furthermore, we added the IRT parameters of both the 1-PL and 2-PL model for each questionnaire in table 5 and rephrased the results section on IRT model fitting as follows:

“Table 5 shows the item parameters for the 1-PL and 2-PL models fitted to the data. S4 Table shows comparisons between the 1-PL, 2-PL, and 3-PL models for each scale. The 1-PL model was the best fitting model for the Generic items, General anaesthesia – II and Regional anaesthesia scales. The 2-PL model was the best fitting model in the General anaesthesia – I, Spinal anaesthesia, Epidural anaesthesia, and PSA scales. Table 5 also shows the item fit per scale for the best fitting model. No item misfit was detected. Fig 2. shows the item characteristic curves (ICC) per scale for the best fitting model.” (lines 368-374)

4. **Remark**: The manuscript will be more representable if it is re-written and reanalyzed. Please consult checklists, for example from Equator network.

**Answer**: We were unable to find a suitable reporting guideline within the Equator network. However, we relied on the papers by Boateng et al and Reeve et al to organize and present our findings. (line 76) We consider these publications to be a solid basis in representing and reporting IRT analyses. As mentioned in our answer on remark #1 we did restructure the paper and incorporated important information that was originally submitted as supporting information into the main manuscript. Furthermore, in line with your remark #7 and remark #28 by Reviewer #2, we made small adjustments to the text to facilitate a better understanding of the progress from the initial item set to the 7 final questionnaires. We believe these revisions, coupled with addressing the remarks of both reviewers makes the manuscript more representable.

“Each selected factor represented a separate scale, corresponding to a separate questionnaire, and was evaluated in the next phase.” (lines 159-160)

“Initially, six knowledge domains were defined: Generic items, General anaesthesia, Spinal anaesthesia, Epidural anaesthesia, Regional anaesthesia and PSA. Then, 51 items covering these knowledge domains were formulated, which were subsequently supplemented by 9 items following the review of a larger group of experts, in total 60 items. The full list of 60 items …” (lines 260-263)

“The RAKQ is a set of seven questionnaires: one generic questionnaire and six questionnaires covering five different anaesthesia techniques, of which general anaesthesia is divided into two questionnaires. In total the RAKQ contains 40 multiple-choice items.” (lines 406-408)

5. **Remark**: S1 & S4, in my opinion, should be in the main manuscript.

**Answer**: Both appendices do provide important information for the reader. We have incorporated them into the main manuscript as Table 1 and Figure 2. Thank you for this suggestion.

**In-text remarks:**

6. **Remark**: Abstract must include summarized results from EFA and IRT

**Answer**: The abstract has been revised to include a summary of the results, as you suggested, with the introduction and conclusion having been shortened to adhere to a 300-word limit.

7. **Remark**: Sub-questionnaires should be renamed to sub-domains, facets or sections.

**Answer**: The term “sub-questionnaires” was intended to refer to the 7 questionnaires of the RAKQ, however, its usage was not consistent. Following your recommendation and a thorough examination of the text, we have revised the language throughout the text to achieve greater uniformity in terminology. We aim to use “domain” when discussing the predefined knowledge domains, “factor” when discussing latent variables explaining patterns of correlation, “construct” when discussing the theoretical concept underlying a scale, “scale” when referring to the instrument that is evaluated in Phase II and “questionnaire” when writing about either the initial set of items presented to the participants, or the final products of the process.

8. **Remark**: Ethical approval should be last paragraph in this section

**Answer**: We moved the paragraph to the end of the Methods section. (lines 245-251)

9. **Remark**: The number of items developed in phase I seem to differ according to the methods and results section.

**Answer**: In the methods section of our manuscript, we utilized the expertise of two researchers/anaesthetists to develop the initial 51 items. This served as the starting point for the development of the scale. In section B, Content Validity, we expanded the pool of experts consulted, which led to the addition of 9 additional items. We acknowledge that this process is not adequately represented in the manuscript and could benefit from further clarification.

We changed the text in the Results section in the following way:

“Initially, six knowledge domains were defined: Generic items, General anaesthesia, Spinal anaesthesia, Epidural anaesthesia, Regional anaesthesia and PSA. Then, 51 items covering these knowledge domains were formulated, which were subsequently supplemented by 9 items following the review of a larger group of experts, in total 60 items. “ (lines 260-263)

10. **Remark**: what is language level B1

**Answer**: B1 is a language level as classified by the Council of Europe in the Common European Framework of Reference for Languages (CEFR). We have added a reference to this paragraph. (Line 103)

11. **Remark**: This should be mentioned clearly as sample size determination for the analysis. In addition, this section is more suitable in Scale Evaluation phase. (Line 114)

**Answer**: Given the fact we did perform EFA and the rule of thumb is to have about 10 completed questionnaires per item, the calculation of participants needed to fulfil this was incorporated into the Scale Development phase. Concurrently addressing remark #26 by reviewer #2, we changed the text in the results and methods section to clarify the number of participants needed given the number of items developed.

In the methods section:

“As a rule of thumb, the sample size for IRT analysis requires ten participants per item [9]. Therefore, the appropriate number of participants was calculated based on the resulting number of items generated during phase I-1. (Item generation).” (lines 113-115)

In the results section:

“Given the fact 60 items were generated during phase I-1 (Item generation), a sample size of 600 participants would be needed, which was approximated by including 577 patients visiting the preoperative outpatient clinic.“ (lines 270-272)

12. **Remark**: My personal view on this step, is that, this step was redundant, because the knowledge domains were decided earlier on during the development. In IRT context, each of this domain can be checked for the IRT parameters of interest and checked for unidimensionality. Oblique rotation is also not necessary, because each knowledge domain is not meant to be correlated and each knowledge domain is meant to stand alone from the start. (Line 132)

**Answer**: We addressed this remark answering remarks #2 and #13.

*Answer to remark #2:* We appreciate reviewer's insight regarding the implementation of Exploratory Factor Analysis (EFA) in our study to identify latent dimensions, particularly in relation to the knowledge scales. While we acknowledge the validity of this perspective, and on which we agree in other circumstances, we would like to explain our rationale for employing EFA in the current study. Our questionnaire was developed from the perspective of an anaesthetist, keeping in mind the patient perspective, but the questions where not developed solely from the patient's perspective. Although we had a notion of the various domains within our questionnaire from the anaesthetist standpoint, we were uncertain whether they truly aligned with patients' perceptions. Through our analysis, we discovered that unidimensionality was not completely evident, especially for the overall anaesthesia set, which, when divided into multiple questionnaires, in our opinion, would serve as a more robust foundation for subsequent Item Response Theory (IRT) analysis, in which it is approached to take into account the patient perspective more accurately.

We added an extra sentence to the third paragraph in the discussion:

“This implies that the generation of items was based on predefined domains formulated by experts. Since this was a first endeavour in constructing a comprehensive knowledge questionnaire on anaesthesia, we conducted exploratory factor analysis to explore underlying latent constructs which could have been less obvious at forehand. During exploratory factor analysis we found some items deemed important had low factor loadings (<|0.4|). This could mean that indeed different underlying constructs can be identified, hindering our efforts to create unidimensional scales within predefined knowledge domains. Nevertheless, based on discussions within the team and the fact that the data was collected from a large multicentre sample [11], we proceeded with a lower threshold (>|0.25|). In future studies, it could be valuable to also include the patient perspective in developing items, which could enhance unidimensionality of the scales.” (line 422-432)

*Answer to remark #13:* We also kindly refer to our answer on remark #2 and elaborate in line with this answer, when considering the current remark (#13). In the analysis phase, we found that the items, contrary to the initial assumption of strict separation, demonstrated interconnectedness. Consequently, we opted to employ oblique rotation to allow for items to correlate to one another within the context of our theoretical framework. For instance, an item from the perspective of an expert could be associated with the construct of "tracheal intubation," while another item could be related to "postoperative care." However, when viewed from the perspective of a patient, both items could be more strongly linked to the construct of "complications". It is thus, that the same knowledge is measured in item level, but the knowledge domains differ based on which perspective is taken into account.

13. **Remark**: Oblique rotation is not in line with unidimensionality assumption.(Line 145)

**Answer**: We also kindly refer to our answer on remark #2 and elaborate in line with this answer, when considering the current remark (#13). In the analysis phase, we found that the items, contrary to the initial assumption of strict separation, demonstrated interconnectedness. Consequently, we opted to employ oblique rotation to allow for items to correlate to one another within the context of our theoretical framework. For instance, an item from the perspective of an expert could be associated with the construct of "tracheal intubation," while another item could be related to "postoperative care." However, when viewed from the perspective of a patient, both items could be more strongly linked to the construct of "complications" and thus correlate. Therefore, the same knowledge can be measured on item level, but the knowledge domains can differ based on which perspective is taken into account.

14. **Remark**: It is also recommended to check using unidimTest() function in ltm package which uses modified parallel analysis. (Line 177)

**Answer**: As recommended by the reviewer, we performed an additional check for unidimensionality using the unidimTest() function from the ltm package. Unidimensionality was shown on all questionnaires. Consequently, we have incorporated this check into both the methods and results sections of our manuscript. Furthermore, we have included an additional figure showcasing the relevant scree plots as supporting information to clarify the findings (S3 Figure).

“The final set of items in each scale was subjected to an additional check for unidimensionality with Modified Parallel Analysis [22].” (lines 178-180)

“The scree plots following Modified Parallel Analysis are presented in S3 Figure in the Online Supporting Information, showing unidimensionality on all scales.” (lines 315-317)

15. **Remark**: “SEm” is highlighted. (Line 218)

**Answer**: We utilized SEm instead of SEM to distinguish between the standard error of measurement and the standard error of the mean. Although both SEM and SE are used to represent the standard error of measurement, SEM seems to be used more commonly. Therefore, we updated our notation from SEm to SEM as suggested. (Lines 220, 221 and 384)

16. **Remark**: Software / packages used should be mentioned here instead of scattered at several places in the text. (Line 229)

**Answer**: We moved the references to software packages to the Statistics and data analysis paragraph.

“Unidimensionality and local independence were assessed with the R package ‘lavaan’ [32] and ‘ltm’[33]. Monotonicity, scalability and invariant item ordering (IIO) were assessed with the R-package ‘mokken’ [21, 34]. Differential Item Functioning (DIF) was evaluated with the R-package ‘lordif’ [35]. Exploratory Factor Analysis, IRT modelling and assessment of the test information were performed with the R-package ‘mirt‘ [23, 36]. “ (lines 234-239)

17. **Remark**: The results can be rearranged into Scale development (which includes item development) and Scale Evaluation. (Line 236)

**Answer**: In line with the remark on the Methods section (remark #1), we rearranged the results into “Scale development” and “Scale Evaluation”, as suggested.

18. **Remark**: This is too short to describe the item + scale development. Qualitative account of the development must be described and elaborated. The items should not be relegated to supplementary material. (line 241)

**Answer**: We acknowledge the importance of this information and have incorporated the supplementary list of items into the main manuscript. The new Table 1 also indicates the reason for removal which contributes to a better understanding of the qualitative account of the development.

“The full list of 60 items, as a non-validated English translation of the Dutch original, that resulted after checking for content and face validity is shown in Table 1. Additionally, this table indicates the phase at which each item was removed from the item set and the reason for its removal.” (lines 263-266)

“In Table 1, the last column indicates the rationale for the exclusion of particular items from the item set.” (lines 304-305)

19. **Remark**: To be combined with item development. (Line 248)

**Answer**: We acknowledge this suggestion. In line with answer on remarks #1 and #17 we have changed this section.

*Answer to remark#1:* We agree with your observation that the development of items and scales must be carried out concurrently, where we may have given the impression that the phases are separate. To accurately convey the message that item and scale development are interconnected, we have modified the headings and Figure 1 as you proposed.

*Answer to remark #17:* In line with the remark on the Methods section (remark #1), we rearranged the results into “Scale development” and “Scale Evaluation”, as suggested.

20. **Remark**: More suitable in Scale Evaluation. (Line 249)

**Answer**: Since we choose to conduct exploratory factor analysis, it was necessary to have a set of responses available during the Scale development phase. See also the answer on remark #11.

21. **Remark**: Again, I find this unnecessary for knowledge scale development. (Line 261)

**Answer**: We kindly refer to our answer on remark #2. Should there be any additional points of consideration or specific areas requiring further attention, we remain open to making necessary adjustments to ensure the utmost clarity and coherence in our work.

*Answer to remark #2:* We appreciate reviewer's insight regarding the implementation of Exploratory Factor Analysis (EFA) in our study to identify latent dimensions, particularly in relation to the knowledge scales. While we acknowledge the validity of this perspective, and on which we agree in other circumstances, we would like to explain our rationale for employing EFA in the current study. Our questionnaire was developed from the perspective of an anaesthetist, keeping in mind the patient perspective, but the questions where not developed solely from the patient's perspective. Although we had a notion of the various domains within our questionnaire from the anaesthetist standpoint, we were uncertain whether they truly aligned with patients' perceptions. Through our analysis, we discovered that unidimensionality was not completely evident, especially for the overall anaesthesia set, which, when divided into multiple questionnaires, in our opinion, would serve as a more robust foundation for subsequent Item Response Theory (IRT) analysis, in which it is approached to take into account the patient perspective more accurately.

We added an extra sentence to the third paragraph in the discussion:

“This implies that the generation of items was based on predefined domains formulated by experts. Since this was a first endeavour in constructing a comprehensive knowledge questionnaire on anaesthesia, we conducted exploratory factor analysis to explore underlying latent constructs which could have been less obvious at forehand. During exploratory factor analysis we found some items deemed important had low factor loadings (<|0.4|). This could mean that indeed different underlying constructs can be identified, hindering our efforts to create unidimensional scales within predefined knowledge domains. Nevertheless, based on discussions within the team and the fact that the data was collected from a large multicentre sample [11], we proceeded with a lower threshold (>|0.25|). In future studies, it could be valuable to also include the patient perspective in developing items, which could enhance unidimensionality of the scales.” (line 422-432)

22. **Remark**: My general comment on this, is that scales should also rely on theoretical and qualitative aspects. These sub-questionnaires were determined by EFA, which I find, from the way it was performed, guided by theoretical and qualitative judgements. I addition, EFA was performed with oblique (correlation) rotation, where orthogonal rotation (no correlation) is a better option if the aim is to look for unidimensional scales. There was no mention of estimation method used for EFA, and where the estimation method is suitable for binary response option. (Line 281)

**Answer**: We kindly refer to our answer on remarks #2 and #13, as we think the answers and revisions in the manuscript relate to the current remark. Given the weight of the remark and for readability we have inserted the answers below.

*Answer to remark #2*: We appreciate reviewer's insight regarding the implementation of Exploratory Factor Analysis (EFA) in our study to identify latent dimensions, particularly in relation to the knowledge scales. While we acknowledge the validity of this perspective, and on which we agree in other circumstances, we would like to explain our rationale for employing EFA in the current study. Our questionnaire was developed from the perspective of an anaesthetist, keeping in mind the patient perspective, but the questions where not developed solely from the patient's perspective. Although we had a notion of the various domains within our questionnaire from the anaesthetist standpoint, we were uncertain whether they truly aligned with patients' perceptions. Through our analysis, we discovered that unidimensionality was not completely evident, especially for the overall anaesthesia set, which, when divided into multiple questionnaires, in our opinion, would serve as a more robust foundation for subsequent Item Response Theory (IRT) analysis, in which it is approached to take into account the patient perspective more accurately.

We added an extra sentence to the third paragraph in the discussion:

“This implies that the generation of items was based on predefined domains formulated by experts. Since this was a first endeavour in constructing a comprehensive knowledge questionnaire on anaesthesia, we conducted exploratory factor analysis to explore underlying latent constructs which could have been less obvious at forehand. During exploratory factor analysis we found some items deemed important had low factor loadings (<|0.4|). This could mean that indeed different underlying constructs can be identified, hindering our efforts to create unidimensional scales within predefined knowledge domains. Nevertheless, based on discussions within the team and the fact that the data was collected from a large multicentre sample [11], we proceeded with a lower threshold (>|0.25|). In future studies, it could be valuable to also include the patient perspective in developing items, which could enhance unidimensionality of the scales.” (line 422-432)

*Answer to remark #13:* In the analysis phase, we found that the items, contrary to the initial assumption of strict separation, demonstrated interconnectedness. Consequently, we opted to employ oblique rotation to allow for items to correlate to one another within the context of our theoretical framework. For instance, an item from the perspective of an expert could be associated with the construct of "tracheal intubation," while another item could be related to "postoperative care." However, when viewed from the perspective of a patient, both items could be more strongly linked to the construct of "complications". It is thus, that the same knowledge is measured in item level, but the knowledge domains differ based on which perspective is taken into account.

23. **Remark**: I find the decision to test out and mix different IRT models difficult to justify here. The decision must be based on why you want to use such model (ie to determine difficulty only assuming same discrimination etc), not because the model fit the data. (Line 335)

**Answer**: We kindly refer to our answer on remark #3.

*Answer to remark #3:* We recognize that in developing a scale based on supposed concepts, those scales should represent the concepts and unidimensionality should be obvious. Furthermore, we subscribe to the remark that when implementing a questionnaire, e.g. when designing a Computerized Adaptive Test (CAT), a theoretical rationale for the choice of the model should be formulated. The 2-PL model provides more opportunities to differentiate between patients with sufficient or insufficient knowledge, with the contribution of the items to the estimation of the knowledge level being different. An argument for using a 1-PL model is the premise that every question is of equal importance in estimating the knowledge level. Given the exploratory nature of this study, we sought to provide information on the quality of the items and different models. When the development of a CAT is undertaken, a choice should be made based on the characteristics of the data as well as on the rationale behind the choice for a 1- or 2-PL model. To convey this message in the manuscript we added the following passage to the discussion:

“Given the exploratory nature of our study, we compared the relative fit of 1-, 2- and 3-PL models to assess which model showed the best fit to the data. The 3-PL models never showed to be an enhancement over the 2-PL models, in line with our approach to reduce the element of guessing by adding an additional response option, “ I don’t know”. It should be noted that when employing the questionnaire, a choice must be made between a 1-PL and 2-PL model. This decision should not only be based on the fit of the model to the data, but also on more fundamental considerations. A 2-PL model provides more opportunities to differentiate between patients with sufficient or insufficient knowledge, with the contribution of the items to the estimation of the knowledge level being different. Conversely, an argument for using a 1-PL model is the premise that every question is of equal importance in estimating the knowledge level.” (lines 448-455)

Furthermore, we added the IRT parameters of both the 1-PL and 2-PL model for each questionnaire in table 5 and rephrased the results section on IRT model fitting as follows:

“Table 5 shows the item parameters for the 1-PL and 2-PL models fitted to the data. S4 Table shows comparisons between the 1-PL, 2-PL, and 3-PL models for each scale. The 1-PL model was the best fitting model for the Generic items, General anaesthesia – II and Regional anaesthesia scales. The 2-PL model was the best fitting model in the General anaesthesia – I, Spinal anaesthesia, Epidural anaesthesia, and PSA scales. Table 5 also shows the item fit per scale for the best fitting model. No item misfit was detected. Fig 2. shows the item characteristic curves (ICC) per scale for the best fitting model.” (lines 368-374)

## Reviewer #2:

24. **Remark**: Suggest adding references for the following:

- Line 55 – “Miller et al.” [3]
- Line 74 – “Dutch Law on Medical Research” (for readers that are not familiar with Dutch regulatory requirements)
- Line 78 – “This study was conducted in compliance with the principles of the Declaration of Helsinki.”

**Answer**: Thank you for your additions, the three references are added. The ethical considerations paragraph was moved to the end of the methods section, in line with a suggestion of Reviewer #1.

25. **Remark**: The number of items selected based on factor loading have been described as both 50 (line 30) and 51 (line 94). Please clarify.

**Answer**: Indeed, the numbers seem not to be in line with each other. However, the "50" and "51" concerns different item sets. The 51 items are the ones that were created as the initial item set by two anaesthetists. Then, a team of experts added 9 items, bringing the total to 60 items. After exploratory factor analysis was conducted on these 60 items, 50 items remained. Some adjustments were made in response to a remark #9 of Reviewer #1 regarding the transition from 51 to 60 items. We have further clarified the numbers by reformulating the original text:

“Based on factor loadings (≥0.25) and considering clinical relevance this initial item set was reduced to 50 items, distributed over the seven domains. Each domain was processed to produce a separate questionnaire. Through an iterative process of item selection to ensure that the questionnaires met the criteria for Item Response Theory modelling, 40 items remained in the definitive set of seven questionnaires.” (line 31-36)

26. **Remark**: Lines 115 to 120 – The authors mention to use 10 participants per item as a rule of thumb when performing an IRT modeling, stating “…which was met by including 577 patients visiting the preoperative outpatient clinic.” While the authors state 510 patients need to be included, it appears from Table 4 that 40 questions were included in the final IRT-model (requiring 400 participants). Additionally, there is some discrepancy in the number participating and those that actually returned survey data. In line 117, the authors mention 577 participants are included. In line 250, the authors indicate that 577 participants completed the questionnaire. In line 251, the response rate is listed at 55% and 28% across the two sites. Line 74 indicates that written informed consent was obtained from all subjects. It is unclear if 577 participants consented to receive the questionnaire, or 577 participants returned the questionnaire, given the response rates listed.
    - Please clarify how many participants were approached, how many participants were consented to receive surveys, and how many participants completed surveys at each site.
    - If 577 participants received the questionnaire and there were response rates of 55% at Erasmus MC (~ 176 participants [0.55*319]) and 28% at ASZ (~73 participants [0.28*258]), please discuss the limitation of not reaching 400 participants needed for the IRT-model.
    - Given the comments above, please adjust the language in lines 250 to 251 to reflect the number of participants that returned surveys; if applicable.

**Answer**: We acknowledge the numbers we provided can be confusing. We revised the text to provide clearer explanations regarding the progression of participants. Moreover, we identified an inaccuracy in the calculation of the response rate for the ASZ sample site and we corrected this. The total number of participants who provided informed consent and completed the questionnaire after being approached was 577, out of a total of 610 patients at the Erasmus MC and 950 at ASZ.

We changed the text into the following to clarify the number of approached patients and actual participants who gave informed consent. Furthermore, we focused on providing information about the number of participants in the results section, while minimizing it in the methods section:

In the methods section:

“As a rule of thumb, the sample size for IRT analysis requires ten participants per item [9]. Therefore, the appropriate number of participants was calculated based on the resulting number of items generated during phase I-1. (Item generation). These participants were recruited at two hospital sites. Between September 2020 and November 2020, participants were included on the outpatient preoperative clinic of the Erasmus Medical Centre (Erasmus MC) in Rotterdam, the Netherlands, a university hospital. From April 2021 to May 2021, participants were included on the outpatient preoperative clinic of the Albert Schweitzer Hospital (ASZ) in Dordrecht, the Netherlands, a general teaching hospital.” (lines 113-120)

In the results section:

“Given the fact 60 items were generated during phase I-1 (Item generation), a sample size of 600 participants would be needed, which was approximated by including 577 patients visiting the preoperative outpatient clinic. In the Erasmus MC, 610 patients consented to be approached regarding this study. The response rate was 55% (n = 336) and of these respondents, 95% provided informed consent for inclusion and completed the questionnaire (n = 319). In ASZ, 950 patients consented to be approached regarding this study. The response rate was 39% (n = 370) and of these respondents, 70% provided informed consent for inclusion and completed the questionnaire (n = 258). “ (lines 270-277)

27. **Remark**: Lines 122 to 125 – The authors indicate that the questionnaire was adjusted after administration to Erasmus MC participants and prior to administration in ASZ participants. Please provide information on the exact changes that were made (minimally in the supplement). It is unclear to the reader if these changes are significant enough to disrupt the homogeneity of the sample across two groups. Additionally, were any of the changes made presented to the original group (Erasmus MC) in a test-retest method to ensure reliability of the instrument? If not, this should be discussed as a limitation.

**Answer**: We agree that the changes made to the items could influence the presented results of the study. Though we carefully considered the changes, it should be clear to the reader what is changed. Therefore, we added an appendix dedicated to the changes in the questionnaire between Erasmus MC and ASZ (S1 Document). The items that were changed, were not presented to the original group (Erasmus MC) afterwards. However, we analyzed the potential differences in item formulation using DIF, as explained in the method section. We therefore changed the text in line 123 to indicate that we paid attention to the altered items by examining differential item functioning (DIF). In line 359 we already wrote that 4 items were flagged for DIF, of which 1 was an item that was altered (GA2). The influence of DIF was considered negligible. Therefore we did not consider this to be a limitation.

“Special attention on differential item functioning was paid to the altered items during the scale evaluation phase.” (line 125-126)

28. **Remark**: Line 371 – The statement “This questionnaire, the RAKQ, contains a set of seven questionnaires covering five different anesthesia techniques and one generic domain…”, is confusing. Is this one questionnaire with seven components/sections, or seven individual questionnaires? Based on previous language I would assume it is 6 sections in which general anesthesia has been divided into 2 components. Please clarify.

**Answer**: We acknowledge that the sentence as it is in the manuscript can be optimized. We revised the text as follows:

“The RAKQ is a set of seven questionnaires: one generic questionnaire and six questionnaires covering five different anaesthesia techniques, of which general anaesthesia is divided into two questionnaires. In total the RAKQ contains 40 multiple-choice items.” (lines 406-408)

We have made several minor adaptations throughout the text to further clarify the subdivision of the RAKQ into 7 questionnaires.

e.g. “After completing the questionnaires, the patients were interviewed about the appropriateness of the questionnaires to measure knowledge …” (lines 107-108) and “Each selected factor represented a separate scale, corresponding to a separate questionnaire, and was evaluated in the next phase.” (lines 159-160) and “The resulting scales from phase I and II each form a separate questionnaire within the RAKQ.”(line 400)

29. **Remark**: Lines 397 to 403 – The challenges and importance of scalability are discussed. In line 399, the authors write, “Improvements in scalability could result in more items concerning less broad subjects. This would lead to lengthier questionnaires that are less user-friendly…”. This is implying potentially adding more items in the future to improve scalability. This is actually contradictory to the methods described in this manuscript where the authors removed items to increase scalability (lines 294 to 333).

**Answer**: The items that were removed in the process of checking scalability were found to be operating on a different construct than the remaining items, which negatively affected scalability. In the discussion, we state that generating additional items that align with the same construct would improve scalability by creating scales that focus on narrower subjects, rather than the broader coverage provided by the current RAKQ questionnaire. However, this approach would require a larger number of questionnaires/scales to achieve the same level of coverage as the current RAKQ questionnaire.

We revised the text into the following:

“Generating additional items that align with the same construct could improve scalability, but could also result in a division of the current scales into more scales, each focusing on a narrower knowledge domain, rather than the broader coverage provided by the current questionnaires in the RAKQ. This approach would consequently require a larger number of questionnaires to achieve the same level of coverage as the current set of RAKQ questionnaires which would impair user-friendliness.” (lines 439-444)

30. **Remark**: Line 418 – The authors describe the potential next steps using computerized-adaptive testing (CAT). While the methods used in this manuscript have been appropriate for developing and validating an instrument, no reliability testing has been performed on the current instrument. Additionally, no description of previous reliability testing on individual items/components that comprise the instrument from the original question sources have been mentioned. Please discuss the need to test reliability prior to developing CAT.

**Answer**: Your comment on reliability testing is valued as it is indeed an important next step before initiating CAT development. We have incorporated the following statement to emphasize this point:

“Furthermore, conducting reliability testing on the validated questionnaires before developing a CAT is warranted to ensure the stability and precision of the IRT parameters across different ability levels.” (lines 481-4836)

31. **Remark**: Lines 128 to 129 – The relationship between the education of anesthetic technique and the administration of the developed instrument lacks clarification:

- Where clinicians instructed to educate participants in any systematic way?
- Were all participants educated on all topics that would be collected, including techniques they would not receive?
- Were participants instructed to answer all questions, or was the questionnaire tailored in which participants were instructed to answer only those sections that applied to their anesthetic technique? If the questions were tailored, was an initial question asking the patient what type of anesthetic technique they would be receiving, asked? Doing so would enhance future CAT development, and should be discussed.

**Answer**: We acknowledge the information we have provided in the manuscript regarding the method and level of education of the participants is limited. Answering your specific questions:

- Clinicians were not instructed to educate participants in a systematic way. They were not informed of the inclusion of the patients in this study, and educated the patients appropriate to the planned surgery, following common clinical practice.
- Participants were only educated on subjects relevant to the planned surgery.
- Participants were instructed to answer all questions, regardless of the anaesthesia technique suited to the planned surgery (line 128).

For this exploratory study, we considered patients undergoing surgery to be a suitable population, as the urgency to gather knowledge on subjects regarding anaesthesia would be higher than the general population. Additionally, the demographics of this population would reflect the target population best. However, future refinement of these questionnaires would certainly include standardization of the education provided to participants.

We added text to the methods section:

“All participants were asked to complete all questionnaires, regardless of the anaesthesia technique they were educated on. Furthermore, the anaesthetists were not informed about the participation of their patients in this study, and the education provided was limited to anaesthesia techniques relevant to the planned surgery and in accordance to standard practice.” (lines 128-132)

We added a paragraph to the discussion:

“Additionally, targeting populations educated on the specific anaesthesia technique assessed in each questionnaire and providing standardized instructions to the anaesthetist educating the patients, would enhance the development of the questionnaires and, consequently, of the CAT. This approach would results in a more sensitive and reliable assessment of the participants’ abilities within the respective domains.” (lines (483-487)

32. **Remark**: Please provide information on completeness of returned surveys at each site (ideally by anesthetic technique).

**Answer**: In table 2 (Demographics and clinical characteristics) we specified the number of patients that underwent certain anaesthetic techniques per sample site. It is important to note that all participants completed the entire survey, including all anaesthesia techniques. As a result, we did not encounter any missing data in the RAKQ items. To emphasize this we added the following text to the results section:

“All participants answered all items in the questionnaire.” (line 279)
